# Toward integration of glycan chemical databases: an algorithm and software tool for extracting sugars from chemical structures

**DOI:** 10.1007/s00216-024-05508-1

**Published:** 2024-08-30

**Authors:** Masaaki Matsubara, Evan E. Bolton, Kiyoko F. Aoki-Kinoshita, Issaku Yamada

**Affiliations:** 1 The Noguchi Institute, Itabashi, Tokyo 173-0003 Japan; 2 National Center for Biotechnology Information, National Library of Medicine, National Institutes of Health, Bethesda, MD 20894 USA; 3 Glycan and Life Systems Integration Center, Soka University, 1-236 Tangi-machi, Hachioji, Tokyo 192-8577 Japan

**Keywords:** Glycan, Structure extraction algorithm, Chemical structure, Database integration

## Abstract

Integration of glycan-related databases between different research fields is essential in glycoscience. It requires knowledge across the breadth of science because most glycans exist as glycoconjugates. On the other hand, especially between chemistry and biology, glycan data has not been easy to integrate due to the huge variety of glycan structure representations. We have developed WURCS (Web 3.0 Unique Representation of Carbohydrate Structures) as a notation for representing all glycan structures uniquely for the purpose of integrating data across scientific data resources. While the integration of glycan data in the field of biology has been greatly advanced, in the field of chemistry, progress has been hampered due to the lack of appropriate rules to extract sugars from chemical structures. Thus, we developed a unique algorithm to determine the range of structures allowed to be considered as sugars from the structural formulae of compounds, and we developed software to extract sugars in WURCS format according to this algorithm. In this manuscript, we show that our algorithm can extract sugars from glycoconjugate molecules represented at the molecular level and can distinguish them from other biomolecules, such as amino acids, nucleic acids, and lipids. Available as software, MolWURCS is freely available and downloadable (https://gitlab.com/glycoinfo/molwurcs).

**Graphical Abstract:**

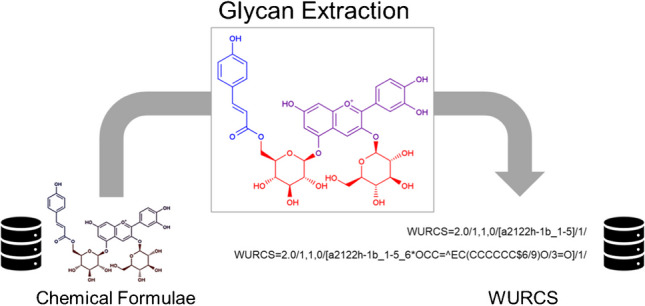

**Supplementary Information:**

The online version contains supplementary material available at 10.1007/s00216-024-05508-1.

## Introduction

Glycans are a class of biopolymers. They are well-known to play important roles in biological processes [1]. While there is no high-throughput sequencing technology widely available yet for glycans due to their structural complexity, sequencing technologies for glycans have been developed in recent years [2], and these efforts have allowed databases to accumulate glycan datasets at an ever increasing rate. However, each dataset (or dataset aggregating database) uses different representations for the glycan structures based on their respective purposes and/or scopes, making cross-integration of data difficult. In particular, there is a huge gap between data systems using biological representations for glycans and those using chemical representations for glycans.

Both biological representations and chemical representations of glycan structures have advantages and disadvantages. Biological glycan representations use trivial names (such as condensed systematic IUPAC names [3]) or use simple symbols (such as SNFG [4, 5]), since many important glycan structures are composed of limited kinds of monosaccharides that can be used to represent them. This approach allows treating many complex glycan structures in simple yet intelligible ways, but makes it difficult to represent uncommon monosaccharides or slight modifications of common monosaccharides. On the other hand, chemical representations, i.e., chemical structural formulae, which use atoms and bonds to represent a chemical structure, can represent every possible monosaccharide, for the most part. However, it is not easy to understand what monosaccharides are included in the all atom/bond chemical representation of the glycan structure because the representation does not explicitly distinguish monosaccharides as structural units.

Thus, we developed WURCS (Web3 Unique Representation of Carbohydrate Structures) [6, 7], a notation for representing any glycan structure in a unique and linear manner, to integrate the representations of and data systems containing glycans. To cover both advantages of biological and chemical representations, WURCS can represent glycans by distinguishing monosaccharides and other moieties (modifications) but also many chemical features using unique atomic notation for monosaccharide backbones and modifications. Together with our development of text format conversion systems between WURCS and other glycan representations, e.g., GlycoCT [8], we were able to integrate many glycan databases and accumulate glycan structures from datasets into GlyTouCan [9], as an archive of unique glycan structures. Leveraging WURCS and GlyTouCan, for example, a large variety of “omics” data related to glycans were integrated and made available through the GlyCosmos Portal [10].

We have also tried to integrate databases that use chemical representations via WURCS. However, this is much more complicated than the case of biological representations. A major reason is that these chemical databases contain glycans as a part of larger chemical compounds and often cover many kinds of (chemist synthesized) carbohydrates and are not limited to biologically important ones. Of course, we must also consider the fact that most known chemical compounds do not contain glycans. Therefore, to integrate glycans in chemical compound databases, it is necessary to appropriately determine whether the target compound contains glycans, and to consider a method to extract only the glycan portion from a chemical structure.

Although the definition of sugars (monosaccharides and glycans) has been discussed for many years, there is still no uniform definition. Even *2-Carb*, which is the nomenclature of carbohydrates recommended by IUPAC-IUBMB [3], only defines the nomenclature of the parent monosaccharides and derivatives and does not discuss to what extent they are considered sugars. Despite this, we have been investigating a method to appropriately identify monosaccharides since we proposed WURCS 1.0 [6]. The most basic idea is to determine whether a carbon chain is appropriate as a monosaccharide backbone based on our own rules, referring to the ideas generally adopted in the nomenclature of organic compounds including carbohydrates. However, the strategy has an essential lack of a sufficiently adequate method to properly assess whether a given carbon chain should be considered as a monosaccharide or not. (More specifically, there were only indicators such as the length of the carbon chain and the ratio of hydroxyl groups present, which is not sufficient to make a determination.) Under these conditions, there were many carbon chains that could be considered monosaccharides but should not be. Thus, we decided to consider a new method to determine the range of allowed carbon chains based on the carbon chain length and the number of modifications, which relies on several requirements. First, it is necessary to avoid (parts of) structures that are not generally treated as glycans, such as amino acids and nucleotides. This condition is required when processing databases dealing with glycoconjugates. Next, known monosaccharides (e.g., those with a biological representation) must be considered as monosaccharides. In particular, at least those monosaccharides defined as SNFG symbols must be considered to be able to link to databases with biological representations. In between these two opposing sides of “black” and “white” (i.e., identifying “non-glycan” from “glycan”), there are many shades of “gray” due to the lack of a formal definition of a glycan.

In this paper, we present an algorithm to satisfy the two primary conditions described above, implemented in the software package MolWURCS. We also discuss the conditions for discriminating between monosaccharides and other parts of a molecule based on an idea of modification count, which shows how much a carbon chain is modified from the standard state of a monosaccharide backbone (as a way to distinguish glycan from non-glycan). MolWURCS is available as an open-source command-line application and as a Java library in GitLab (https://gitlab.com/glycoinfo/molwurcs). The standalone command-line application reads chemical structures in MOL, SDF, and SMILES formats and outputs the glycan structures found within a chemical structure in WURCS format. It can also read WURCS and write the corresponding chemical structure in MOL, SDF, or SMILES format. The input and output formats can be switched using the provided options. MolWURCS has been tested on a set of monosaccharides with trivial names defined in IUPAC and SNFG, amino acids comprising proteins, nucleic acids comprising DNA/RNA, and several types of lipids.

## Methods

### Algorithm

The algorithms of MolWURCS are implemented in Java 1.8 with the support of the Chemistry Development Kit (CDK) [11] version 2.8. The code and Maven repository are freely available in GitLab (https://gitlab.com/glycoinfo/molwurcs). MolWURCS extracts glycans from chemical compounds and exports them as WURCS, which may have only glycan moieties (default) or glycans with an aglycone (by specifying the option “--with-aglycone”). The entire workflow can be separated into three primary steps: molecular preprocessing, glycan extraction, and WURCS export. While the basic overview of these processes was initially proposed in our WURCS 1.0 paper [6], it has been extended to include our new glycan extraction algorithms. Therefore, in this section, we will discuss the changes from the previous effort and describe the workflow broadly. Particular emphasis will be placed on more refined algorithms for the glycan extraction processes.

### Molecular preprocessing

In this step, analyses are performed on the input molecules and those out of scope are removed from further consideration. The removals are necessary to only allow molecular elements that can be handled according to the WURCS normalization procedure presented in the WURCS 1.0 paper [6]. For example, a free-radical atom is not supported in WURCS. The results of the analyses will be used during subsequent processing steps. It is worth noting that these processes were updated to be supported by the CDK toolkit (see Section S3 in the Supplementary Information).

### Glycan extraction

This step has two primary parts: carbon chain extraction and modification extraction. The purpose of this is to identify which atoms and bonds are part of the glycan and then to further identify common (or allowed) modifications to a glycan. Structures that cannot pass this processing step do not contain a glycan and no WURCS is output.

### Carbon chain extraction

This processing step extracts the carbon chains corresponding to any monosaccharide backbones that may be present. Since monosaccharides have a wide variety of derivatives, it is necessary to allow a certain extent of carbon chains to be regarded as the monosaccharide backbones. Thus, we defined the reference structures which are central for defining the range of allowed carbon chains and developed a method to determine the range of allowed carbon chains based on the carbon chain length and the number and type of modifications. Table 1 shows the parameters and conditions for determination of the allowed carbon chains.

**Table 1:** Current and previous (WURCS 1.0) default conditions for each structural feature of the carbon group to use upon carbon chain extraction

Structural feature	Current condition	Previous condition (WURCS1.0)
The number of carbons	Between 5 and 12 (containing carbons on branch)	Between 3 and 9 (not containing carbons on branch)
The number of branches	Up to 1	Not limited
Containing or bonding to a carbocycle	Not allowed for either containing or bonding	Allowed bonding, but not containing
Containing or bonding to a π-cyclic atom	Not allowed for either containing orbonding	Allowed bonding, but not containing
Forming a small (3- or 4-membered) cyclic ether	Not allowed	Not allowed
Modifications on carbons	Not allowed when the “modification count” is exceeded, where the threshold value is determined by the number of carbons	At least 2 carbons must have a hydroxyl group or *O*-linked substituent, and at least 2 carbons must have a substituent linked with N, O, or S atom

These conditions were determined based on the length and the modifications of carbon backbones of known monosaccharides as reference structures, from simple cases, such as hexoses and pentoses, to more complicated ones, such as sialic acids and branched monosaccharides. The interpretation of the carbon chain was extended to carbon groups from the previous conditions to cover branched carbon chains. The conditions for the number of carbon atoms and branches are determined to take known branched monosaccharides but also to eliminate highly complicated structures from further consideration. While the conditions of carbon cyclic and π-cyclic atoms are based on the previous approach, this is now extended to exclude the carbon chains to which they are connected. This extension is effective in eliminating non-monosaccharide structures, such as nucleotides and aromatic amino acids which contain aromatic rings or π-cyclic atoms, and cyclitols and sterols which contain carbocycles. The condition for eliminating 3- or 4-membered cyclic ethers is considered as these are normally considered as an intermediate and are exceedingly rare as a monosaccharide element. Furthermore, the previous conditions using the number of carbons connecting N, O, and S atoms were refined and developed into a new condition using “modification count.”

The “modification count” is considered to determine how far the carbon chain is from the basic state of a monosaccharide backbone, which has a (potential) carbonyl group at position 1 or 2 and one hydroxyl group on each of the other carbon atoms (such as aldose or 2-ketose). There are five different types of modifications considered. Replacement type (*N*_replacement_) accounts for deoxy and replacements of hydrogen, hydroxyl or carbonyl group. Unsaturation type (*N*_unsaturation_) accounts for unsaturation (double or triple bond) of the carbon chain. These two modification types are counted for each individual carbon atom. Ring type (*N*_ring_) accounts for anhydro and lactone rings, while extra carbonyl type (*N*_extra_carbonyl_) accounts for extra (potential) carbonyl groups, and anomer penalty type (*N*_anomer_penalty_) provides a penalty for an uncommon or no anomeric position. The total of these is obtained as the “modification count” of the carbon chain (*N*_mod_), as shown in Eq. (1). Basically, the further and rarer the modification is from the standard state of monosaccharide, the higher the count.

**Fig. 1:**
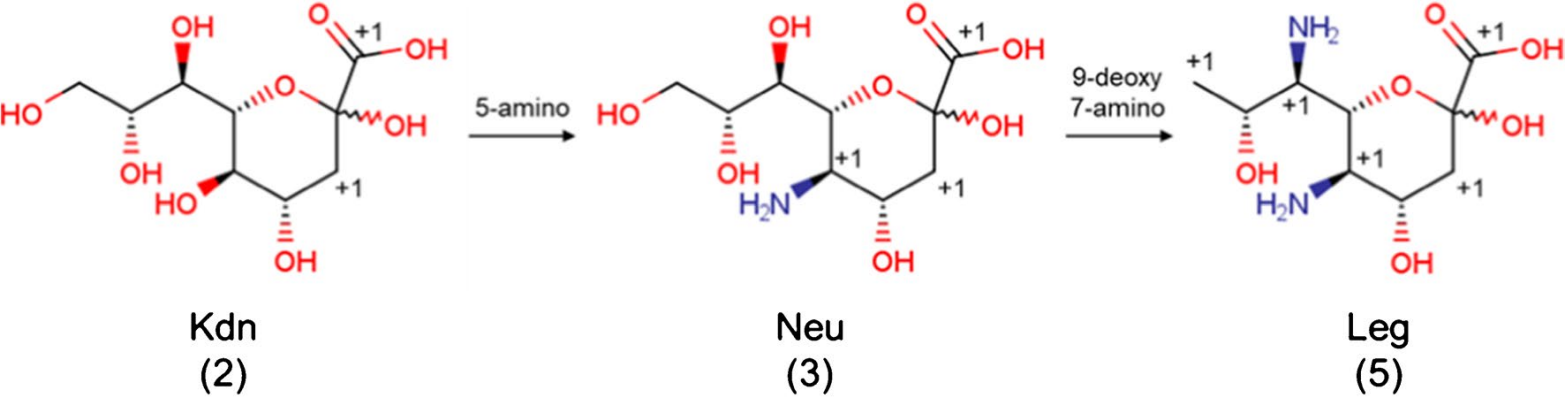
Structures for 3-deoxy-d-*glycero*-d-*galacto*-non-2-ulopyranosonic acid (Kdn) and two of its derivatives, 5-amino-3,5-dideoxy-d-*glycero*-d-*galacto*-non-2-ulosonic acid (Neu) and 5,7-diamino-3,5,7,9-tetradeoxy-d-*glycero*-d-*galacto*-non-2-ulopyranosonic acid (Leg). The number in parentheses under the name of each structure is the respective modification count. The “+1” depicted next to a given atom indicates a modification count increase due to a change in the structure away from an “ideal” monosaccharide (such as glucose)

To exclude carbon chains far from the basic state of a monosaccharide, we set a threshold value *θ*(*n*_c_) for the modification count, which is determined from the number of carbon atoms in the carbon group *n*_c_. The default *θ*(*n*_c_) value is *n*_c_ - 3, meaning a modification count greater than three indicates a carbon backbone that is unlikely to be a glycan monosaccharide. For carbon backbones 9 or more carbon atoms, a maximum threshold value of 5 is set. In other words, longer carbon chains can only have a total of 5 changes from the normal (typical) glycan monosaccharide before it is excluded from consideration (see Fig. 1 for an example of three monosaccharides (Kdn, Neu, and Leg) and their respective modification counts).

1 $${N}_{\text{mod}}={\sum }_{i}^{{n}_{\text{c}}}\left({N}_{\text{replacement}}^{i}+{N}_{\text{unsaturation}}^{i}\right)+{N}_{\text{ring}}+2{N}_{\text{extra}\_\text{carbonyl}}+{N}_{\text{anomer}\_\text{penalty}}$$

2 $$\theta \left({n}_{\text{c}}\right)=\text{min}( {n}_{\text{c}}-3, 5 )$$

More detailed considerations for these conditions are described in Sections S4 and S5 in the Supplementary Information.

After satisfying the modification count check, the main chain for each carbon group is selected. The priority for the selection of the main chain is determined by the following three factors:

- With anomer: The carbon chain has an anomeric carbon (such as the case of a cyclic hemiacetal or hemiketal).
- With potential anomer: The carbon chain has no anomeric carbon but has carbonyl group(s).
- With longer chain: The carbon chain length is longer than the others.

For example, a carbon chain of a cyclic hemiacetal or hemiketal is preferred to one having aldehydic or ketonic carbonyl group but no cyclic hemiacetal or hemiketal, and their carbon chains are preferred to one without carbonyl group, such as an alditol. If these conditions are the same, the longer carbon chain is preferred. Note that only the main chains are treated as backbone carbon chains and the branched chains are treated as the modification moieties based on the WURCS definition where only the linear carbon chain is treated as a monosaccharide backbone.

### Modification extraction

In this process, the atom groups composed of connected atoms which are not included in the carbon chains are extracted as modifications. Although these modifications are basically treated as part of the glycan, such as substituents of monosaccharides and/or linkages between monosaccharides, the aglycone portion of glycosides and another molecules (glycosyl acceptors) in glycoconjugates are not regarded as part of the glycan. Thus, we also defined conditions for determining such non-glycan modifications as follows:

- A modification connecting to only anomeric atoms of monosaccharide carbon chains is considered a non-glycan modification
- A modification containing non-organic atoms is considered to be a non-glycan modification
- A modification with branches above a certain threshold (by default, 5 or more) is considered a non-glycan modification
- A modification containing an SSSR ring of a size above a certain threshold (by default, 10-membered ring or larger) is considered a non-glycan modification

According to the basic definition of aglycones of glycosides or glycosyl acceptors of glycoconjugates, modifications connected to only anomeric atoms are regarded as non-glycan modifications. Furthermore, we added some conditions to determine modifications which should not be a part of glycans. Chemically modified glycans are often found in chemical compound databases. However, the modified part should not (necessarily) be regarded as a part of the “glycan.” Therefore, we set a condition to exclude modification parts with non-organic atoms. The conditions for the number of branches and the ring size of the SSSR (the smallest set of smallest rings) were determined for excluding macrocyclic atomic groups such as macrolides and other complex atomic groups which are not connected via a glycosidic linkage.

The modifications which meet these conditions are excluded, and then all atoms that connect the modifications on the backbone carbon chain are replaced by oxygen, regardless of what the element is.

Although the excluded modifications will not be output as WURCS by default, these exclusions can be turned off when the “--with-aglycone” option is specified.

## Results

### Detection of monosaccharides with trivial names

In order to extract all of the monosaccharides with trivial names, we applied our glycan extraction algorithm to all of the monosaccharides listed in the Appendix of *2-Carb*. In this test, we focused on modification counting rules to confirm how the rule works to distinguish carbon chains. Here, stereoisomeric monosaccharides were treated as the same structure because our rule does not consider the stereochemistry of the structures. As a result, we assembled a dataset of 66 monosaccharides without stereochemistry.

**Fig. 2:**
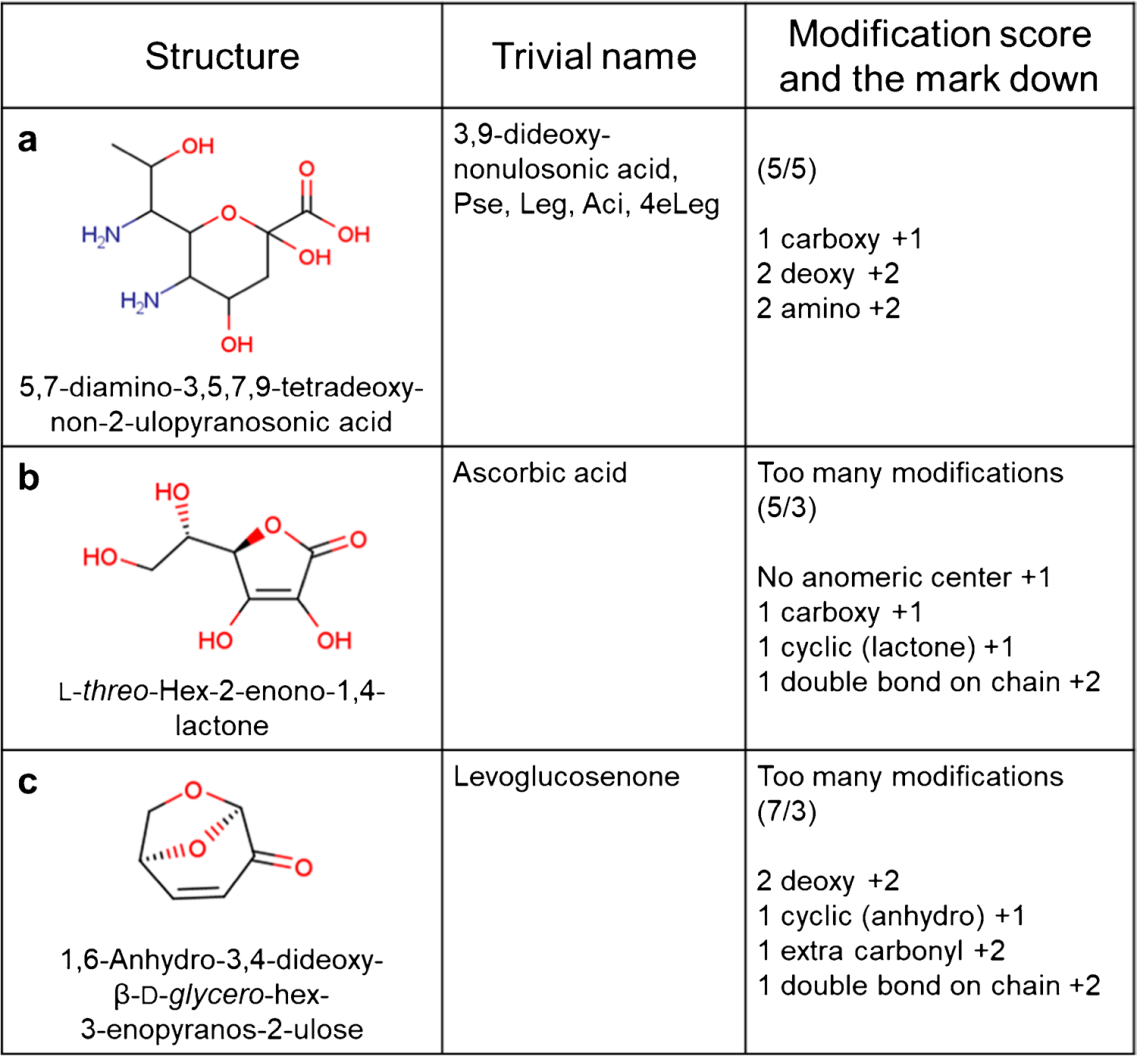
Structures and modification counts of **a** 5,7-diamino-3,5,7,9-tetradeoxy-non-2-ulopyranosonic acid, **b** ascorbic acid, and **c** levoglucosenone. The structure of **a** has the highest modification count (+5) of the monosaccharides with SNFG symbols. The structures of **b** and **c** are not regarded as monosaccharide due to too many modifications

To check that our main purpose of detecting all monosaccharides with SNFG symbols as monosaccharides is achieved, we first focused on 23 of the 66 in our dataset. While one of them, which subsumes 3,9-dideoxy-nonulosonic acid and its stereoisomers (Fig. 2a), has the highest modification count (+5) which is on the border of the maximum threshold value for nonoses, it can still be considered as a monosaccharide using this algorithm. The other 22 were also confirmed to have modification counts below the thresholds. Therefore, at least all of the monosaccharides with SNFG symbols were considered as monosaccharides by our modification counting rule as expected.

Regarding the other 43 structures in this curated validation dataset, 31 have modification counts lower than the thresholds. Of the remaining twelve, seven are trioses or tetroses and are excluded because they are too small to be considered as monosaccharides. The remaining five are ascorbic acid (Fig. 2b), levoglucosenone (Fig. 2c), isolevoglucosenone, forosamine, and purpurosamine C. They are excluded due to having too many modifications. The major reason for ascorbic acid is that an anomeric center is replaced with a lactone. This simple replacement adds +3 to the modification count (i.e., lack of anomeric center and addition of a lactone ring, containing both a ring and a carboxyl group). Although the ascorbic acid could be regarded as a monosaccharide, lactone is one of the features that should not be contained in monosaccharides. Therefore, we do not take ascorbic acid as a monosaccharide. Levoglucosenone has more modifications than the other monosaccharides. Even if it is derived from d-glucose by the loss of three molecules of water, the structure is already far from the basic state of the monosaccharide backbone. Therefore, we do not consider levoglucosanone as a monosaccharide either. Since Isolevoglucosenone has almost the same structure as levoglucosenone, the same decision applies. Forosamine and purpurosamine C are also not taken as monosaccharides because they have too many deoxy and amino modifications. These examples help to emphasize the limits of this approach but, when considered from the perspective of larger glycans (made up of cross-linked monosaccharides), it would appear to strike the necessary balance to cover known glycans (as opposed to all synthetically possible monosaccharides).

The modification counts and the details for all structures in our dataset are shown in Section S6 in the Supplementary Information.

### Exclusion of structures comprising biopolymers other than glycans

To confirm that our rule excludes structures comprising biopolymers other than glycans, we considered nucleotides and amino acids. In summary, while an additional exclusion rule for a carbon chain connecting to aromatic atom groups was needed to exclude the nucleotides, no special rule was needed to exclude amino acids. Here, we show the application of our algorithm to the major amino acids.

**Fig. 3:**
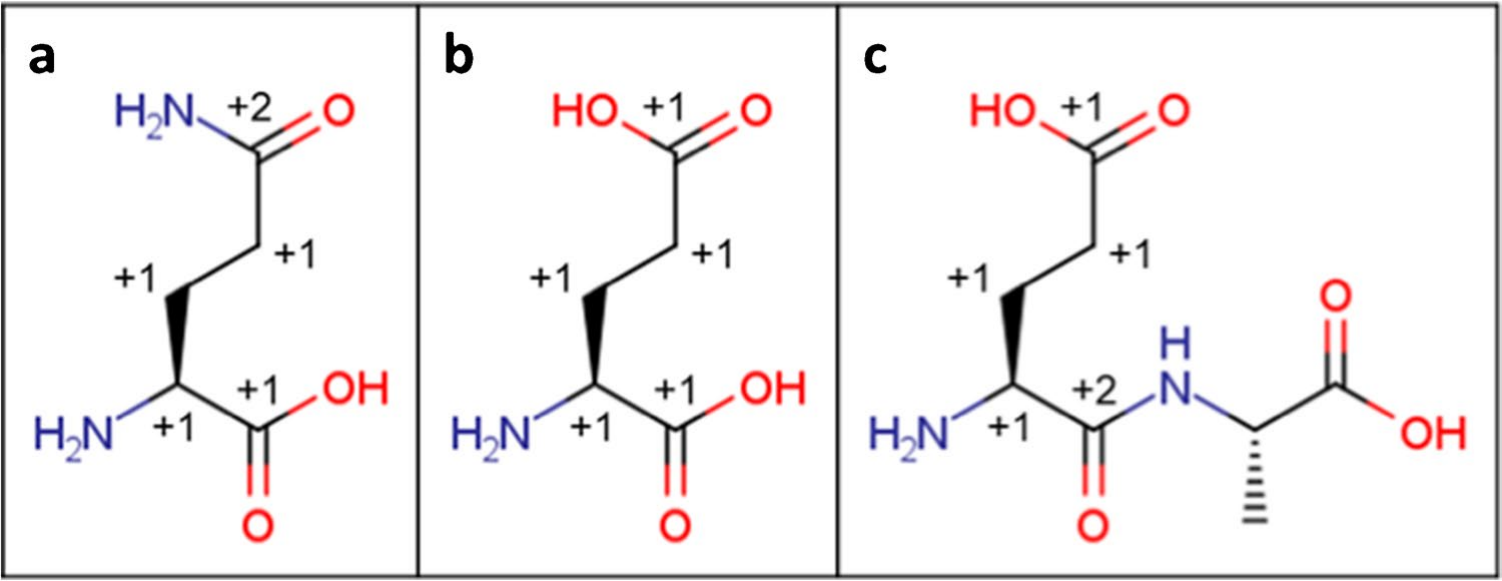
Structures of **a** glutamate, **b** glutamic acid, and **c** glutamylalanine, which are not regarded as monosaccharides. The numbers are modification counts on the individual carbon atoms. Each amino acid except for alanine has enough carbon atoms but too many modifications to be considered as a monosaccharide. Forming peptide bonds further increases the modification count (carboxyl with +1 to amide with +2)

For the 20 most common amino acids comprising proteins, all of them can be excluded because eight (including Ara, Asn, Asp, Cys, Gly, Met, Ser, and Thr) do not have enough carbon atoms (less than 5 carbon atoms in the carbon chain), four (including His, Phe, Trp, and Tyr) are aromatic amino acids, and eight (including Arg, Gln, Glu, Ile, Leu, Lys, Pro, and Val) have enough carbon atoms but too many modifications as a monosaccharide, such as deoxy and replacement of hydroxyl groups. Applying modification count to Gln and Glu is shown in a and b of Fig. 3. The other amino acids with different carbon length, such as β- and γ- amino acids, can also be excluded because the additional carbon is not in the basic state (deoxy) and thereby the modification count is only increased. Moreover, the amino acids forming peptide chains are excluded as well because forming peptide bonds changes the carboxyl group to amide and thereby the modification count is further increased by +1 (Fig. 3). Therefore, our algorithm can readily exclude most known naturally occurring amino acids and peptides.

### Glycan extraction from LIPID MAPS Structure Database

Next, we investigated that our glycan extraction algorithm can extract only glycan parts from glycoconjugates.

There are various types of glycoconjugates in which a glycan is bound to other types of molecules. The most typical glycoconjugates are glycoproteins, which are proteins with glycans connected with glycosidic bonds. While the type of amino acid residue to which the glycan is attached is defined (Asn for *N*-linked glycans and Ser or Thr for *O*-linked glycans), it is already verified in the previous section that these are not considered as monosaccharides. Furthermore, our algorithm removes the non-monosaccharide part connected via glycosidic bonds. Therefore, it is clear that our algorithm can extract only glycan parts from glycoproteins. On the other hand, other glycoconjugates such as glycosides and glycolipids have a great variety of molecules to which sugars (monosaccharides and larger glycans) are attached. Therefore, they are suitable for validating our algorithm.

The LIPID MAPS Structure Database (LMSD), a service provided by LIPID MAPS (LIPID Metabolites And Pathways Strategy), is a structural database that contains many such glycolipids and glycosides [12]. Notably, all registered lipid structures are classified based on their comprehensive classification criteria, LIPID MAPS Lipid Classification System [13]. The categories (or classes) of glycolipids and glycosides are also present in that classification, and it is possible to determine from the categories and classes whether or not sugars are present in these structures.

Thus, we applied our glycan extraction algorithm to all structures registered in the LMSD to determine whether it adequately extracts only sugar structures based on their categories and (sub)classes. Also, even if some structures in the LMSD contain sugars, all of them should have a lipid at least in part. Therefore, we can also check that our algorithm properly and correctly excludes structures other than sugars through this examination. [Note that all the analysis was performed using our newly developed MolWURCS command-line application (https://gitlab.com/glycoinfo/molwurcs#wurcs-from-molecules), with the SDF file of LMSD structures (obtained on May 2, 2023) as input and WURCS as output.]

### Glycan detection from structures explicitly marked as containing sugars

To confirm whether or not our algorithm extracts sugars from larger structures containing them, we applied our algorithm to all structures in the categories/classes explicitly marked as containing sugars.

The categories and classes that are explicitly marked as containing sugars are the following: fatty acyl glycosides [FA13] (243 entries), glycosylmonoradylglycerols [GL04] (24 entries), glycosyldiradylglycerols [GL05] (101 entries), glycosylglycerophospholipids [GP14] (17 entries), glycerophosphoinositolglycans [GP15] (338 entries), Glycerophosphoethanolamine glycans [GP21] (44 entries), neutral glycosphingolipids [SP05] (2106 entries), acidic glycosphingolipids [SP06] (1375 entries), basic glycosphingolipids [SP07] (1 entry), amphoteric glycosphingolipids [SP08] (1 entry), and Saccharolipids [SL] (1345 entries), where the square brackets indicate the ID of each category or class. As a result of applying our extraction algorithm to the 5595 structures in these categories/classes, sugars could be extracted from all of them. This result indicates that our algorithm is able to extract glycans from structures that are supposed to contain glycans, as it should.

On the other hand, some of the structures classified in the other classes also contain sugars (Table 2). In particular, over 40% (2964 out of 7147) of the polyketide category, which had the largest number of cases, were determined to contain sugars. While this is because most of the structures of the flavonoid class, the majority of polyketide category, exist as glycosides [14], it also demonstrates that our algorithm automatically reveals such a fact as well.

**Table 2:** Comparison between the number of entries in each LIPID MAPS category and the number of entries from which sugars were extracted by our algorithm

	Number of entries			
	LM entries marked as containing sugars by LM		LM entries NOT marked as containing sugars by LM	
LIPID MAPS (LM) categories	Total number for category	Glycans extracted	Total number for category	Glycans extracted
Fatty acyls [FA]	243	243	10313	46
Glycerolipids [GL]	125	125	7616	2
Glycerophospholipids [GP]	399	399	9620	1
Sphingolipids [SP]	3483	3483	1050	107
Sterol lipids [ST]	0	0	3568	391
Prenol lipids [PR]	0	0	2391	226
Saccharolipids [SL]	1345	1345	0	0
Polyketides [PK]	0	0	7147	2964
Total	5595	5595	41795	3737

### Non-sugar removals from LMSD

To verify whether our algorithm properly excludes non-sugars, we examined structures in the LMSD whose entire structure was extracted as a sugar.

Since the LMSD is a database of lipids, all structures in the LMSD must contain lipids. Therefore, if there is an entry whose entire structure is considered as a sugar by our algorithm, it is possible that our algorithm is incorrect. On the other hand, it is also possible that the structure can be considered both lipid and sugar, and we will check that here as well.

**Fig. 4:**
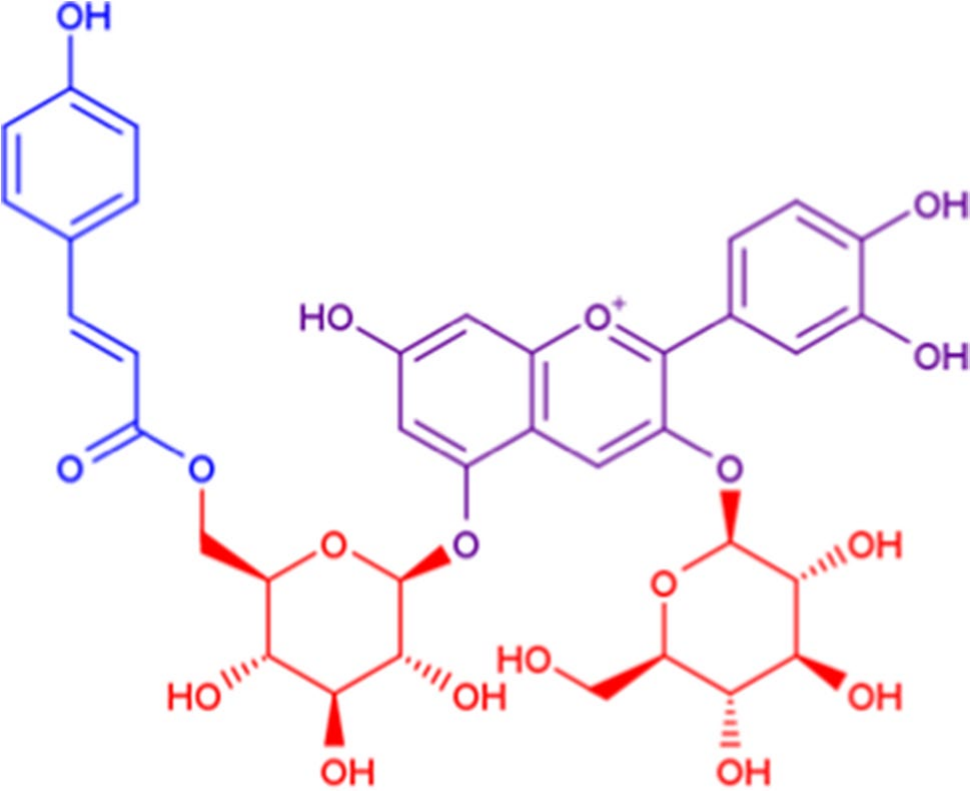
Discrimination result of monosaccharide (red), substituent (blue), and aglycone (purple) moieties for cyanidin 3-glucoside-5-(6-p-coumaroylglucoside) [LMPK12010158] using our algorithm. Two WURCS are output, one being glucose monosaccharide and the other being 6-p-coumaroylglucose, which is glucose with a p-coumaroyl group as substituent. The cyanidin is excluded as an aglycone

As an example of when our algorithm excluded non-sugar moieties, we show the result of a flavonoid glycoside, cyanidin 3-glucoside-5-(6-p-coumaroylglucoside) (Fig. 4). Two monosaccharides, glucose and 6-p-coumaroylglucose, were extracted from this structure as a result of the cyanidin group being excluded as a non-sugar moiety. In contrast to cyanidin, the p-coumaroyl group remained as a substituent because it did not meet the exclusion conditions; i.e., the p-coumaroyl group does not have 4 or more branches and it is connected to a monosaccharide with a glycosidic linkage unlike the cyanidine group.

Of all LMSD structures, there were 594 cases where the entire structure was determined to be a sugar (Table 3). Of these, 581 contained fatty acyl chains as substituents. This means that the lipid moiety was considered a substituent rather than a monosaccharide but did not meet the conditions as a non-sugar moiety (complex structure or structure linked only by glycosidic bonds). Such structures were more common in saccharolipids (546 cases). This is because the saccharolipids have monosaccharides with fatty acyl(s) as its backbone. Since the fatty acyls usually have a simple structure and connect with non-glycosidic bonds, they are not removed as a non-sugar moiety. As such, the saccharolipids are special structures as lipids and this fact may also be a problem when integrating databases of glycans and the lipids. We will discuss this in detail in the “Discussion” section.

**Table 3:** The number of LMSD entries where the entire lipid structure was determined to be a sugar with a breakdown by category

Category	Number of entries		
	With acyl chain	No acyl chain	Total
Fatty acyls [FA]	8	13	21
Glycerophospholipids [GP]	27	0	27
Saccharolipids [SL]	546	0	546
Total	581	13	594

**Fig. 5:**
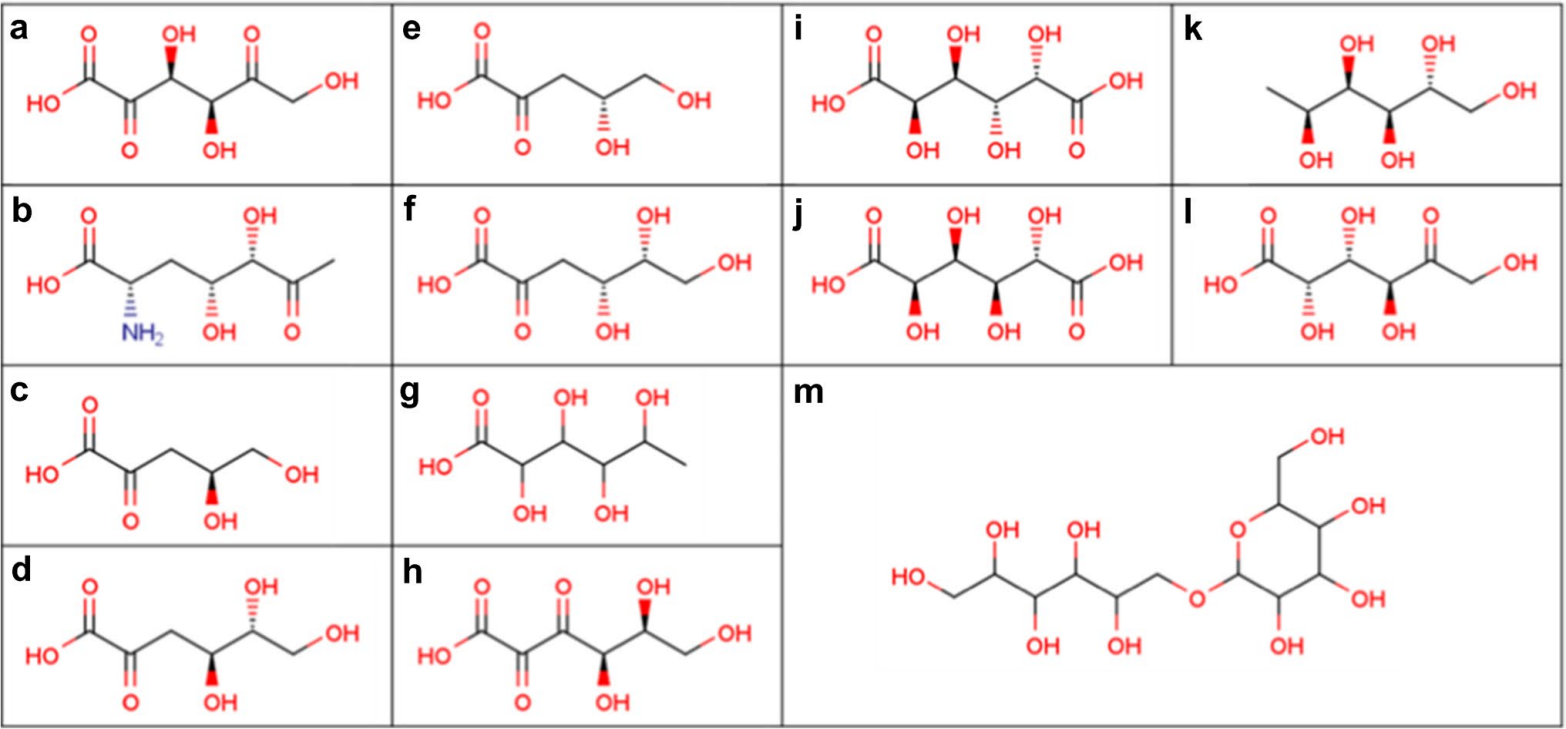
The 13 LMSD structures where the whole structure is regarded as a sugar by our algorithm: **a** 2,5-didehydro-d-gluconic acid [LMFA01050471]; **b** 2-amino-2,3,7-trideoxy-d-*lyxo*-hept-6-ulosonic acid [LMFA01050473]; **c** 2-dehydro-3-deoxy-d-arabinonic acid [LMFA01050474]; **d** 2-dehydro-3-deoxy-d-gluconic acid [LMFA01050475]; **e** 2-Dehydro-3-deoxy-l-arabinonic acid [LMFA01050476]; **f** 2-keto-3-deoxy-d-gluconic acid [LMFA01050486]; **g** fuconic acid [LMFA01050532]; **h** diketo-gulonic acid [LMFA01060195]; **i** galactaric acid [LMFA01170107]; **j** glucaric acid [LMFA01170108]; **k** 1-Deoxy-d-glucitol [LMFA05000598]; **l** d-tagaturonic acid [LMFA05000654]; and **m** 1-*O-*D-Hexopyranosyl-d-hexisitol [LMFA13010063]

The remaining 13 cases ([LMFA01050471], [LMFA01050473], [LMFA01050474], [LMFA01050475], [LMFA01050476], [LMFA01050486], [LMFA01050532], [LMFA01060195], [LMFA01170107], [LMFA01170108], [LMFA05000598], [LMFA05000654], and [LMFA13010063]) had a structure that could be considered a sugar (Fig. 5). The fact that the common names are systematic names for monosaccharides suggests that LMSD (or their data contributor) also considers the structures to be sugars. Twelve of the 13 (Fig. 5a-l) were sugar alcohols and acids within the 5 to 7 chain-length and with no or few modifications. From our modification counting algorithm, their carboxyl groups do not strongly affect the determination for monosaccharide likeness because they are treated as semi-standard states. Moreover, the carbonyl group at position-2 is regarded as a potential anomeric group, which is not counted as a modification. Therefore, these structures cannot be excluded as lipids (non-monosaccharides) by our algorithm. The remaining one (Fig. 5m) is mostly a reduced disaccharide. From the subclass name of this structure (fatty acyl glycosides of mono- and disaccharides [FA1301]) and the fact that the hexopyranose moiety is obviously a monosaccharide, it seems that the hexitol moiety is treated as a lipid.

## Discussion

### Distinction of sugar moieties in saccharolipids

**Fig. 6:**
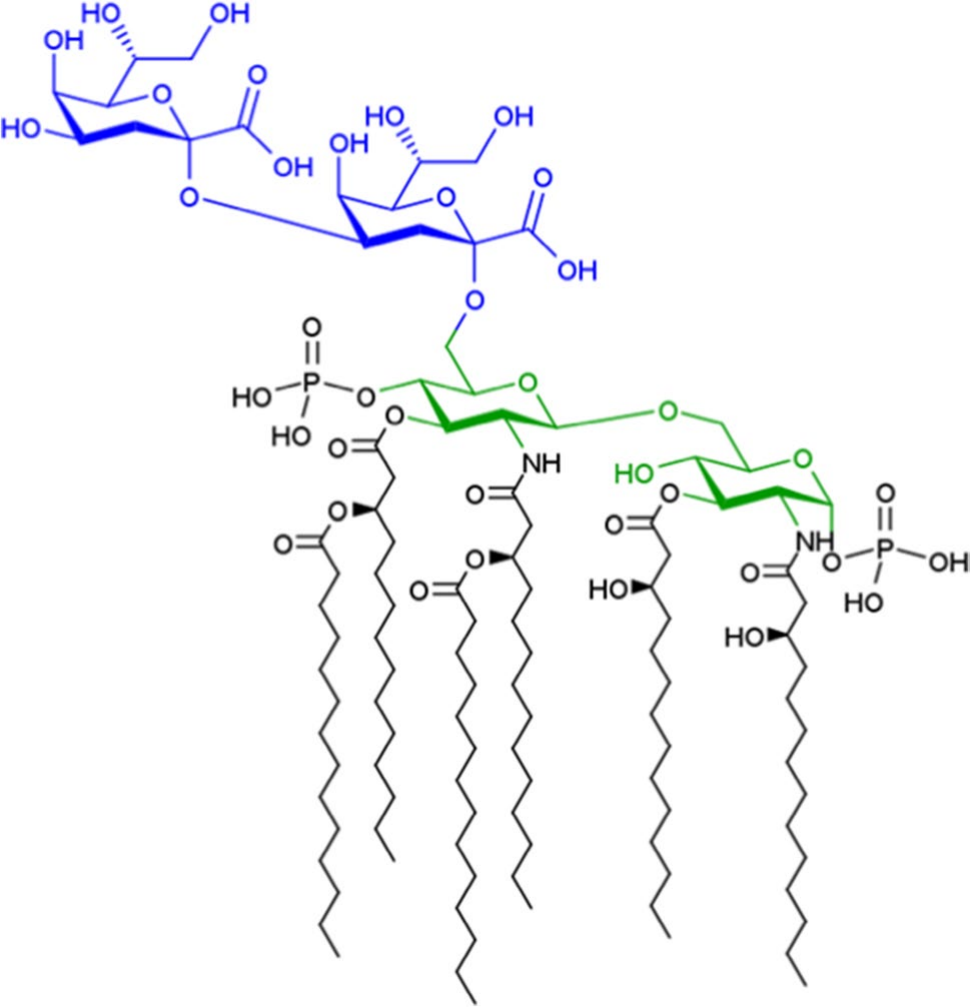
Structure of the saccharolipid, Kdo2-lipidA. Glucosamine residues are in green. Kdo residues are in blue. Acyl chains and phosphate groups are in black. Although the green part is a backbone of lipid A, both green and blue parts are considered as monosaccharide residues by our algorithm

As we described in the “Results” section, based on our rules, saccharolipids are regarded as containing monosaccharide residues, and the other parts, such as fatty acyls and phosphates, are regarded as substituents. However, although the fatty acyls should not be regarded as a glycan part when linking with lipid databases, our glycan extraction rule should not have a classification rule only for the fatty acyls. Furthermore, a part of the sugar moieties in saccharolipids may be better to be considered a lipid part because they serve as a backbone of the lipid. In fact, the minimal lipopolysaccharide required for growth in *Escherichia coli*, Kdo2-lipid A, is a hexa-acylated disaccharide of glucosamine that is glycosylated with two Kdo residues (Fig. 6) [15], and the glucosamines are considered as part of lipid A, leaving the Kdo residues to be a part of the inner core of the lipopolysaccharide. Moreover, saccharolipids are distinct from glycolipids as the glycolipids are defined by IUPAC to have the sugar bound by a glycosidic linkage to a fatty acyl [16]. Thus, LIPID MAPS defines saccharolipids as a distinct category of lipids [17].

Therefore, although we need to discuss with lipid researchers (about which part of a molecule should be considered lipid and which part sugar), if the sugar moieties in saccharolipid should be excluded from the glycan part, we would need to modify this algorithm approach. For example, a new rule to exclude monosaccharides might be: any carbon chain cannot have a long hydrocarbon chain as a substituent other than on the anomeric carbon.

### Comparison with other algorithms

In the field of natural products research, it is necessary to identify sugars but from a completely opposite motivation to ours. For example, in the study of natural products, sugars are present as substituents in their targets, and in many cases, they need to be removed because they can be an obstacle to structural analysis. As software developed to satisfy such requirements, Sugar Removal Utility (SRU) [18] employs algorithms for more general chemical compounds. Germane to this work, SRU identifies the cyclic or linear carbon backbones individually and removes those that meet the requirements as monosaccharides. Since each sugar detection and removal can be used separately, the detection algorithm can be used for glycan extraction. However, this algorithm has limitations in that monosaccharides with long side carbon chains, such as sialic acids, are recognized as different sugars in the cyclic and side chain portions. While this is not a problem in terms of their goal of deglycosylation, it will be a problem in our goal of extraction of glycans and differentiation of monosaccharide elements. Also, SRU cannot detect each of the monosaccharides in cyclodextrin due to the circular sugar detection algorithm, but MolWURCS can. The other difference is that SRU uses predefined patterns for the monosaccharide backbone. While the predefined pattern has the advantage of clearly defining the target structure, it has the disadvantage of not covering the diversity of target structures. Even if SRU allows the addition of predefined patterns by modifying the source code, it is necessary to add the structures one by one. Therefore, our algorithm of “modification count” is better suited for the purpose of extracting a variety of monosaccharide backbones because it allows us to specify multiple structures together, including derivatives, without using predefined patterns.

On the other hand, unlike the linear sugar detection algorithm of SRU, our algorithm does not recognize a part of the carbon chain. The reason for this is that there are only a very limited number of cases in which a part of the carbon chain is considered to be a monosaccharide. Rather, there is a risk of recognizing a part of a carbon chain as a monosaccharide when it should be recognized as a larger structure, such as a macrolide. Therefore, our algorithm targets the whole carbon chain, not just a part of it.

In addition, SRU can detect *C*-glycosidic bonds, which have the glycosidic oxygen replaced by a carbon, but MolWURCS does not, as our algorithm regards the bond as a part of the carbon chain. However, most *C*-glycosidic bonds found in natural products, such as pseudouridine, mangiferin, and barbaloin, have a carbon in the aromatic ring or carbon cyclic which violates our definition, and thereby the carbon chain with *C*-glycoside will end up being excluded from the candidates. The other possible case which should be considered is that the *C*-glycosidic bond is chemically synthesized. However, in most cases, it is not regarded as “glycosides” because it is not hydrolysable unlike the other glycosides. In fact, IUPAC discourages the use of names based on “*C*-glycoside” terminology [19]. Therefore, the *C*-glycosides are not taken into account by our algorithm.

## Conclusion

We presented an algorithm for extracting glycan moieties from molecular structures represented in general chemical representation. This algorithm improves upon the previous WURCS 1.0 effort [6]. The most prominent features in our glycan extraction algorithm are rules for determination of the ranges of candidate carbon chains and modifications. Using these elaborated and unique rules allows us not only to extract known monosaccharides and their derivatives. Limits to modifications prevent atom groups which should not be included as a part of glycans, such as glycosyl acceptors of glycoconjugates.

Our new algorithm was tested using known monosaccharides, such as those which have trivial names or SNFG symbols, but was also tested using counter examples of non-sugar molecules including amino acids and nucleotides. As the results show, all monosaccharides with SNFG symbols are regarded as monosaccharides and amino acids and nucleotides are not, as expected. Moreover, the available structures in the LIPID MAPS Structure Database (LMSD) were used to test the ability of MolWURCS to discriminate between sugar and non-sugar moieties of our algorithm. All structures in the classes explicitly marked as containing sugars were confirmed by our algorithm to contain sugars as they should.

The MolWURCS command-line application embodies the new algorithm and can be used to extract monosaccharide and glycan structures from several molecular formats, such as MOL, SDF, and SMILES, and exports glycans as WURCS, or reads WURCS and exports the glycan structures into molecular formats.

## Supplementary Information

Below is the link to the electronic supplementary material.

Supplementary file1 (DOCX 700 KB)
