# Supplementary material for: Toward integration of glycan chemical databases: an algorithm and software tool for extracting sugars from chemical structures
Source: Anal Bioanal Chem. 2024 Aug 30;417(5):945–56. doi: 10.1007/s00216-024-05508-1 (PMC11782307; doi:10.1007/s00216-024-05508-1)
Supplement: Supplementary file 1 — Supplementary file1 (DOCX 700 KB) [file 216_2024_5508_MOESM1_ESM.docx]

Supplementary Information for

**“Toward Integration of Glycan Chemical Databases:
An Algorithm and Software Tool
for Extracting Sugars from Chemical Structures”**

Authors:

Masaaki Matsubara^1^, Evan E Bolton^2^, Kiyoko F. Aoki-Kinoshita^3^, Issaku Yamada^1^

Affiliations:

^1^The Noguchi Institute, Itabashi, Tokyo, 173-0003, Japan.

^2^National Center for Biotechnology Information, National Library of Medicine, National Institutes of Health, Bethesda, MD 20894, USA.

^3^Glycan and Life Systems Integration Center, Soka University, 1-236 Tangi-machi, Hachioji, Tokyo 192-8577, Japan.

*E-mail: issaku@noguchi.or.jp

List of Contents

[S1. Definition of terms 4](#_Toc167811332)

[S2. MolWURCS Workflow 5](#_Toc167811333)

[S2.1. Glycan extraction workflow 5](#_Toc167811334)

[S2.1.1. Molecular preprocessing 5](#_Toc167811335)

[S2.1.2. Carbon chain extraction 5](#_Toc167811336)

[S2.1.3. Modification extraction 5](#_Toc167811337)

[S2.1.4. Glycan construction 5](#_Toc167811338)

[S2.1.5. WURCS export 6](#_Toc167811339)

[S3. Molecular preprocessing 7](#_Toc167811340)

[S3.1. Molecular normalization 7](#_Toc167811341)

[S3.1.1 Metal atom removal 7](#_Toc167811342)

[S3.1.2 Isotope information removal 7](#_Toc167811343)

[S3.1.3 Charge removal 7](#_Toc167811344)

[S3.1.4. Explicit Hydrogen Removal 7](#_Toc167811345)

[S3.2. Molecular analysis 8](#_Toc167811346)

[S3.2.1. Atom type perception 8](#_Toc167811347)

[S3.2.2. Stereochemistry calculation 8](#_Toc167811348)

[S3.2.3. Carbon cyclic detection 8](#_Toc167811349)

[S3.2.4. Π-Cyclic detection 8](#_Toc167811350)

[S4. Conditions for extractions 9](#_Toc167811351)

[S4.1 Carbon group collection 9](#_Toc167811352)

[S4.1.1. The number of carbons 9](#_Toc167811353)

[S4.1.2. The number of branches 10](#_Toc167811354)

[S4.1.3. Cyclic atom groups 10](#_Toc167811355)

[S4.1.4. Modification count 10](#_Toc167811356)

[S4.2 Carbon chain selection 10](#_Toc167811357)

[S4.3 Modification extraction 10](#_Toc167811358)

[S4.3.1. Only connecting anomeric carbons 11](#_Toc167811359)

[S4.3.2. Non-organic atoms 11](#_Toc167811360)

[S4.3.3. The number of branches 11](#_Toc167811361)

[S4.3.4. The ring sizes of SSSR 11](#_Toc167811362)

[S5. Rules for counting modifications on backbone carbon chain 13](#_Toc167811363)

[S5.1. Definitions of basic states and the modifications 13](#_Toc167811364)

[S5.2. Counting modifications by type 14](#_Toc167811365)

[S5.2.1. Replacements of functional groups 14](#_Toc167811366)

[S5.2.2. Unsaturated bonds of carbon chain 16](#_Toc167811367)

[S5.2.3. Rings bridging carbon on a carbon chain 17](#_Toc167811368)

[S5.2.4. Replacements of heteroatoms on cyclic hemiacetal/hemiketal carbon 17](#_Toc167811369)

[S5.2.5. Extra (potential) carbonyl groups 18](#_Toc167811370)

[S5.2.6. Penalty for (potential) carbonyl groups on uncommon position 18](#_Toc167811371)

[S5.3. Threshold value for modification counts 18](#_Toc167811372)

[S6. Dataset for detection of monosaccharides with trivial names 19](#_Toc167811373)

[S6.1. Monosaccharides with SNFG symbols 19](#_Toc167811374)

[S6.2. Monosaccharides with trivial name (no SNFG symbol) 24](#_Toc167811375)

# S1. Definition of terms

- Sugars
  - Glycans and monosaccharides
- Glycan
  - Two or more monosaccharides connecting with modifications
- Monosaccharide
  - A backbone carbon chain with connecting modifications
- Backbone carbon chain
  - A carbon chain to be monosaccharide backbone
  - Our rule defines the range of the carbon chain which allowed to be regarded as a monosaccharide backbone
- Carbon group
  - A connecting carbon atom group to be candidate of backbone carbon chain
  - Backbone carbon chain is selected as main chain of the carbon group
- Modification
  - A connecting atom group other than backbone carbon chain
  - Our rule defines the range of the modification which allowed to be a part of monosaccharide or glycan

# S2. MolWURCS Workflow

## S2.1. Glycan extraction workflow


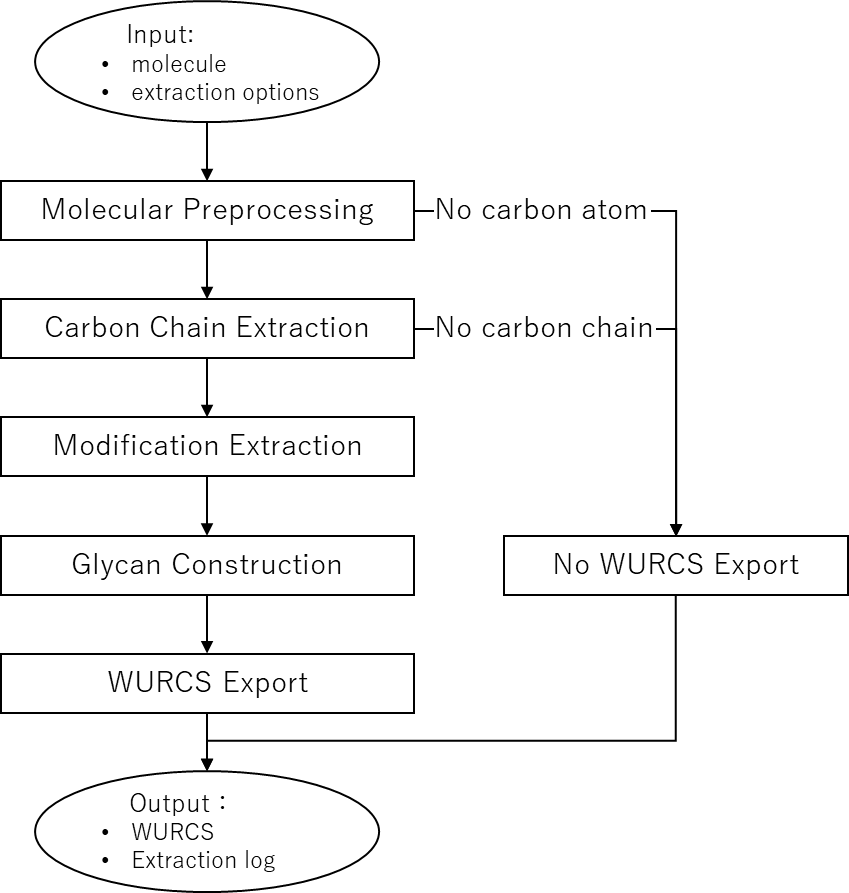


### S2.1.1. Molecular preprocessing

In this phase, some preprocesses necessary for the following extraction and WURCS export are performed. See section S3 in detail.

### S2.1.2. Carbon chain extraction

The first step of glycan extraction. In this phase, carbon chains to be monosaccharide backbone are extracted according to some conditions. The conditions are described in section S4 in detail.

### S2.1.3. Modification extraction

The second step of glycan extraction. In this phase, modifications to be a part of monosaccharide or glycan are extracted according to some conditions. The conditions are described in section S4 in detail.

### S2.1.4. Glycan construction

The final step of glycan extraction. In this phase, connection relationships between extracted carbon chains and modifications are confirmed and then monosaccharide or glycan is constructed by them.

### S2.1.5. WURCS export

The extracted monosaccharide or glycan is converted to WURCS according to the encoding rule. No WURCS is exported when no carbon atom or carbon chain is found or a process is failed.

# S3. Molecular preprocessing

At this process, some of the removals and the analyses are performed for input molecules. The removals are to normalize molecular elements according to WURCS normalization procedure presented in WURCS 1.0 paper. The results of analyses will be used at subsequent processes. The processes were updated from previous ones to be supported by the CDK classes.

This processing will be terminated when any of the processes at here is failed, and then no result is output. Note that a free radical cannot be read because it is not supported in WURCS.

## S3.1. Molecular normalization

In current version of WURCS, metal atoms, isotopes, and charges are not handled, and hydrogens are not represented explicitly. Thus, to simplify the subsequent analyses and to normalize some tautomers, their unused molecular elements are removed or ignored from input molecules before starting glycan extraction and WURCS export.

### S3.1.1 Metal atom removal

Metal atoms are removed in this phase not to consider them as part of a glycan structure.

If a removed metal atom is connected to the other non-metal atoms, the connections are treated as ones to hydrogens.

The following elements are considered as non-metal atoms in this system:

H, He, C, N, O, F, Ne, P, S, Cl, Ar, Se, Br, Kr, I, Xe, Rn, B, Si Ge, As, Sb, Te, At

This determination is according to specification in the CDK class *Elements*.

### S3.1.2 Isotope information removal

Isotope information is ignored even if it is explicitly specified. More precisely, any isotope information is not used in all subsequent processing.

### S3.1.3 Charge removal

Charges are removed in this phase even if it is specified explicitly. With the neutralization of charge by this process, implicit hydrogen attachments or detachments occur for the atoms in ionic state, e.g. -NH_3_^+^ is to be -NH_2_ and -COO^−^ is to be -COOH. However, if the attachments or detachments are not applicable due to delocalization of the charge or the presence of atoms beyond their normal valence, the charges are maintained. Nevertheless, bonds represented as ionic bonds are neutralized to nonionized forms after changing the bond valence. For instance, when a nitro group is represented as “-N^+^(=O)-O^−^”, it is neutralized to “-N(=O)(=O)”.

### S3.1.4. Explicit Hydrogen Removal

All of explicit hydrogens are removed in this phase to normalize the molecular condition and to simplify following process. After this removal, implicit hydrogens will be added for each atom using the CDK class *CDKHydrogenAdder*. Note that atom type perception must be performed before using the class.

## S3.2. Molecular analysis

Some processes need some molecular structural information. Here, we describe the molecular analysis for obtaining the necessary information.

### S3.2.1. Atom type perception

Each atom is perceived its atom type using the CDK class *CDKAtomTypeMatcher*. This process is necessary to add implicit hydrogens.

### S3.2.2. Stereochemistry calculation

Stereochemistry for each atom or bond is calculated using the CDK class *StereoElementFactory*. Calculated stereochemistry is labeled as CIP descriptor to each atom or bond using the CDK class *CIPTool*. The labels will be used in WURCS export.

### S3.2.3. Carbon cyclic detection

Any of atoms contained in carbon cyclic are detected using the CDK class *Cycles*. The detected carbons are used to filter carbon chains containing carbon cyclic.

π

### S3.2.4. π-cyclic detection

Any of atoms contained in π-cyclic (or aromatic ring) are detected using the CDK class *Cycles*.

Here, “π-cyclic” means a ring which all of the contained atoms have electrons. The detected atoms are used to filter carbon chains containing or connecting to the π-cyclic atoms.

# S4. Conditions for extractions

To extract structures suitable for sugars, we determined some conditions for each structural element. Here, we describe the conditions for carbon group, carbon chain and modification.

## S4.1 Carbon group collection

we defined the reference structures that are the central for the range of allowed carbon chains and developed a method to determine the range of allowed carbon chains based on the carbon chain length and the number of modifications.

The following conditions are to take the allowed carbon chains which:

- The number of carbon atoms is between 5 and 12

- The number of branches is up to 1

- No carbon cyclic is contained in

- No π-cyclic atom is contained in or connected to

- No small (3 or 4 membered) cyclic ether is formed

- The “modification count” is not exceeded the threshold value determined by the number of carbon atoms

### S4.1.1. The number of carbons

In our rule, a monosaccharide must have 5 to 12 carbon atoms.

For the minimum number of carbons, Nomenclature of Carbohydrates recommended by IUPAC-IUBMB (*2-Carb*) says that “parent monosaccharides are polyhydroxy aldehydes or polyhydroxy ketones with *three or more carbon atoms*”. However, the most of monosaccharides with three or four carbon atoms (trioses and tetroses) have many characteristics that not monosaccharides. For example, many of them usually do not form cyclic hemiacetals or hemiketals and thereby cannot form glycosidic bond. Moreover, they are often found from various compounds other than monosaccharides, e.g., glycerol of glycerolipids. So that, we do not take trioses and tetroses as monosaccharides.

For the maximum number of carbons, there is no reference in *2-Carb* or the other nomenclatures. Generally, nonoses (e.g., sialic acids) are the longest monosaccharides without branch on the carbon chain, but some of the branched-chain monosaccharides have more than nine carbon atoms. For example, Caryophyllose, a 4-*C*-branched monosaccharide identified from Gram-negative bacteria, has 12 carbon atoms (6 in main chain and 6 in branched chain). So that, we take monosaccharides with up to 12 carbon atoms.


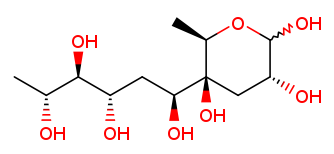


**Fig. S4-1** Caryophyllose.

### S4.1.2. The number of branches

Our rule allows branched-chain monosaccharides since there are some monosaccharides with trivial name. Especially, apiose, a branched-chain pentose, is defined as a SNFG symbol. However, all monosaccharides with trivial name listed in Appendix of *2-Carb* do not have two or more branches. Therefore, we determined that carbon chains with two or more branches are not considered as monosaccharide backbones.

### S4.1.3. Cyclic atom groups

The conditions of carbon cyclic and π-cyclic are used to exclude the carbon chains to which those are connected. These are effective to eliminate non-monosaccharide structures, such as nucleotides and aromatic amino acids which contain aromatic ring or π-cyclic, and cyclitols and sterols which contain carbon cyclic. The condition for eliminating 3 or 4 membered cyclic ether is considered as well because it is normally as intermediate and rare as monosaccharide element.

### S4.1.4. Modification count

The “modification count” is considered to count how much the carbon chain is far from basic state of monosaccharide backbone. The detailed information is described in section S5.

## S4.2 Carbon chain selection

After the carbon chain collection, the main chain for each carbon group is selected. The priority for the selection of main chain is determined by the following factors:

- With anomer

- With potential anomer

- With longer chain

Here, “with anomer” means that the carbon chain has anomeric carbon and “with potential anomer” means that the carbon chain has no anomeric carbon but carbonyl group(s). i.e., a carbon chain of a cyclic hemiacetal or hemiketal is preferred to one having aldehydic or ketonic carbonyl group but no cyclic hemiacetal or hemiketal, and their carbon chains are preferred to one without carbonyl group, such as an alditol. If these conditions are the same, the longer carbon chain is preferred. Note that only the main chains are treated as backbone carbon chains and the branched chains are treated as the modification moieties based on the WURCS definition which only linear carbon chain is treated as monosaccharide backbone.

## S4.3 Modification extraction

WURCS has rule to represent various chemical structures. However, it does not refer to what chemical structures are suitable as a part of glycans. Especially for substituents, there is no clear rule for identifying itself in many cases, and thereby they can become too much large and complicated. Thus, we defined some conditions for excluding the unsuitable substituents.

### S4.3.1. Only connecting anomeric carbons

This condition is based on general definition of aglycone of glycoside and/or non-carbohydrate part of glycoconjugate. Normally, when an atom group which is not considered as monosaccharide and it connected only anomeric carbons, the atom group is regarded as glycosylated aglycone part.

### S4.3.2. Non-organic atoms

In general, chemical modifications make glycan structures diverse and complicated. Thus, we consider that chemical modifications should not be contained in the glycan substituents. There are many discussions about "what are chemical modifications", but at this point, we defined that chemical modifications contain non-organic atoms. Since there are some discussions about "organic atoms" as well, we also defined that the following elements are organic atoms: H, C, N, O, P, S.

These are basically elements contained in natural organic compounds. Although some elements, e.g., F, can be considered to be a part of substituent because many compounds registered in chemical compound databases has them, they are used as some chemical modifications, e.g., labeling, in many cases. Therefore, we limit organic elements more strictly.

### S4.3.3. The number of branches

This condition is for measuring complexity of the chemical structures, i.e., more branches, more complex. Here, we consider that "branch" is an atom with three or more connections to heavy atoms. The number of branches is to be a number of the branched atoms, i.e., atom with three connections has a branch and one with four connections has two branches. Note that the element of branched atom is not limited to carbon currently. Therefore, for example, P of phosphate and S of sulfate are also counted as branched atoms. At this point, we determined that up to four branches are allowed in a substituent. This is considered from the list of substituents described in SNFG document to keep major substituents. On the other hand, we dare not to use "the number of atoms" for filtering because some substituents can be large but have fewer branch, e.g., lipids.

### S4.3.4. The ring sizes of SSSR

This condition is to filter substituents with large ring. Here, the "maximum ring size" means the ring size of a biggest ring of SSSR (smallest set of smallest rings). This is to distinguish single rings from the fused rings. The condition for the number of branches is also effective to filter polycyclic compounds because they have many branches as well. Therefore, we do not have to consider about ring size of polycyclic compounds.

Returning to the "maximum ring size", we need to consider about how much ring size is too large. Basically, we consider that at least the macrocyclic compounds should be excluded as a part of glycans.

The "macrocycles" also has various definitions, but the many of those say that a ring of ten or twelve atoms is on the border line.

Thus, we determined that any substituents in a glycan must not have a ring of ten or more atoms in the SSSR.

# S5. Rules for counting modifications on backbone carbon chain

## S5.1. Definitions of basic states and the modifications

To determine whether or not the carbon chains are suitable as monosaccharide backbones, we defined “basic state” and “modification count”. They are considered to count how much the carbon chain is far from basic monosaccharide backbone, which has a carbonyl group at position 1 or 2 and one hydroxyl group on each of the other carbons, i.e., aldose or 2-ketose. From this definition, hydroxyl group and hydrogens on an alcohol, and carbonyl group and hydrogen on an aldehyde or a ketone are considered as basic state. Since most monosaccharides exist as cyclic hemiacetals or hemiketals, the structures are also basic state. Carboxyl group is an important and common functional group of monosaccharides but usually treated as a modification. Thus, we consider a carboxyl group as a semi-basic state which +1 extra modification count is applied as penalty. The other functional groups are considered as modifications. **Fig. S5-1** shows basic and semi-basic states for terminal, non-terminal and branched carbons.


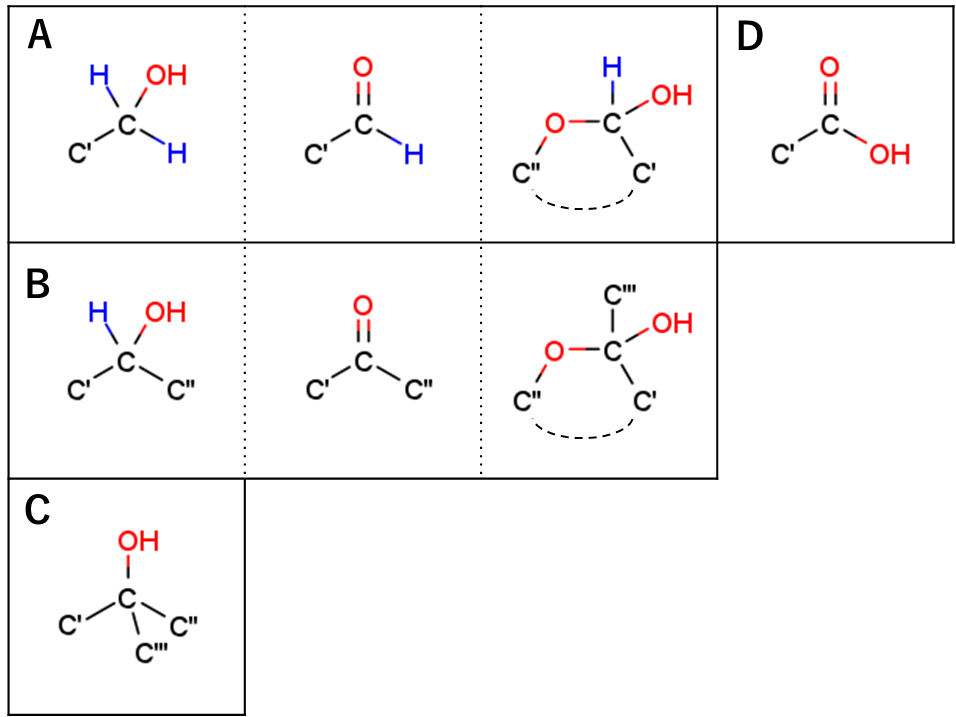


**Fig. S5-1** Basic states for (**A**) terminal, (**B**) non-terminal, and (**C**) branched carbons. For the terminal and non-terminal carbons, not only alcohol but also carbonyl and cyclic hemiacetal/hemiketal (potential carbonyl) groups are treated as basic states. Carboxy group (**D**) is semi-basic state for terminal carbon which originally has +1 modification count.

## S5.2. Counting modifications by type

The modifications are counted individually in a unique manner according to their type, classified as ones of a carbon, including deoxy and replacements of hydrogen, hydroxyl or carbonyl group (*N*_replacement_) or unsaturation (double or triple bond) of carbon chain (*N*_unsaturation_), or ones of a carbon chain, including anhydro and lactone rings (*N*_ring_), extra (potential) carbonyl groups (*N*_extra_carbonyl_), or uncommon or no anomeric position (*N*_anomer_penaly_), and the total of these is obtained as modification count of the carbon chain (*N*_mod_), as shown in Eq. (1). Basically, the further and rarer the modification is from the standard state of monosaccharide, the higher the count.

$$\begin{aligned} \boldsymbol{N}_{\text{mod}}\boldsymbol{=}\sum_{\boldsymbol{i}}^{\boldsymbol{n}_{\text{c}}} \left( \boldsymbol{N}_{\text{replacement}}^{\boldsymbol{i}}\boldsymbol{+}\boldsymbol{N}_{\text{unsaturation}}^{\boldsymbol{i}} \right)\boldsymbol{+}\boldsymbol{N}_{\text{ring}}\boldsymbol{+}{\boldsymbol{2}\boldsymbol{N}}_{\text{extra\_carbonyl}}\boldsymbol{+}\boldsymbol{N}_{\text{anomer\_penalty}}\boldsymbol{\#}\left( SEQ Equation \backslash* ARABIC 1 \right) \end{aligned}$$

### S5.2.1. Replacements of functional groups

In our rule, a modification can be rephrased as a replacement of functional group from basic state. Since the functional groups of the basic states are a hydroxyl group, a hydrogen, and a carbonyl oxygen, we first defined the modification counts for the replacements of those as +1, +2 and +3, respectively. These counts are determined based on their replaceabilities. A deoxy is treated as a replacement from a hydroxyl group to a hydrogen, and thereby counted as +1. When two or more hydroxyl groups are connected, one is regarded as the one of basic state and the other ones are regarded as replacements of hydrogens. Therefore, the second and the latter hydroxyl groups are counted as +2 for each. The modifications connecting with double bond is regarded as replacement of carbonyl oxygen, and thereby counted as +3. For the modification with triple bonds, the basic states do not have them. Thus, we defined that the modifications are combination of replacement of a hydroxyl group and two hydrogens, and thereby counted as +5. A carboxyl group originally has +1 modification count because it is a semi-basic state. Therefore, the modifications on the carboxyl group are counted from the modification count +1 in the same way of the other replacements. Note that any replacements of functional groups must be counted from the basic state. e.g., hydrogens on deoxy state cannot be replaced again because one of the hydrogens is regarded as a modification which is already replaced from a hydroxyl group. Also note that any replacements of a hydrogen on hydroxyl group is not counted as modification because the replacements do not change state around the backbone carbons. **Fig**. **S5-2** shows relationships between modification counts and replacements of functional groups from basic states.


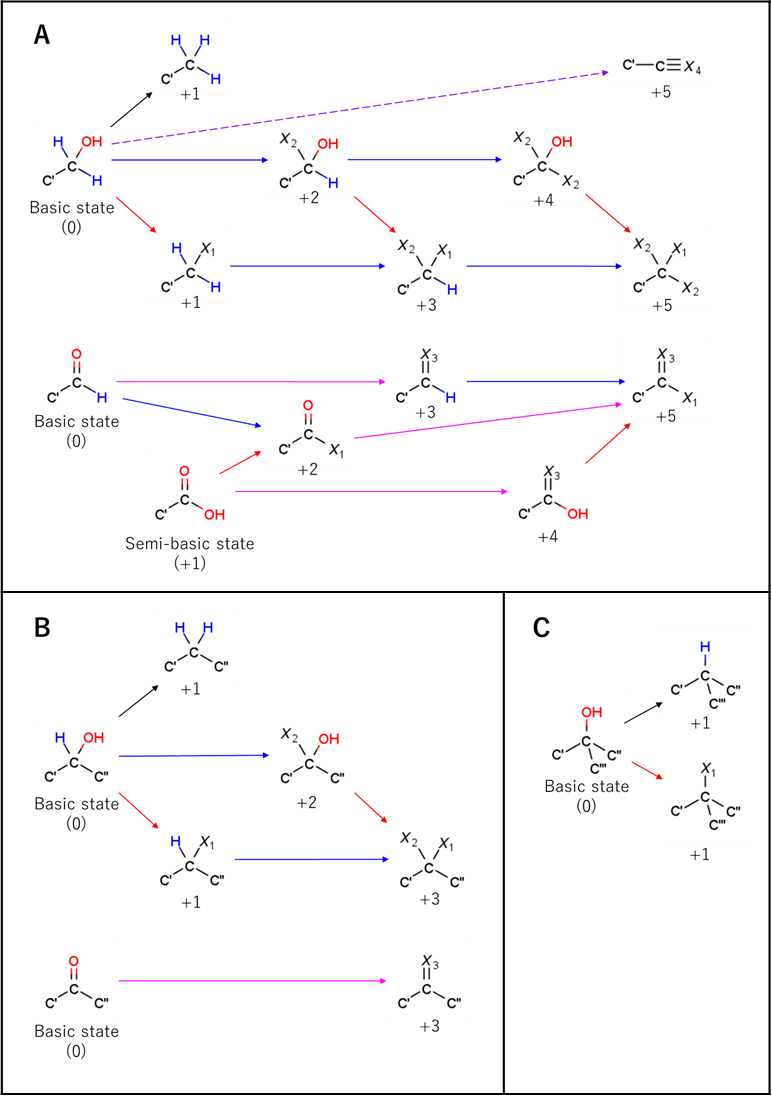


**Fig. S5-2** Relationships between modification counts and replacements of functional groups from basic states on (**A**) terminal, (**B**) non-terminal, and (**C**) branched carbons. The replacements are categorized into deoxy (black arrow, +1), replacement of hydroxyl group (red arrow, +1), replacement of hydrogen (blue arrow, +2) and replacement of carbonyl oxygen (pink arrow, +3). A modification with triple bond is regarded as replacement of hydroxyl group and two hydrogens (purple dashed arrow, +5). Here, *X_1_* is a modification except for hydrogen, hydroxyl group or the derivative which hydrogen on hydroxyl group is replaced to the other modification, including *O*-glycosidic bonds. *X_2_* is not hydrogen. *X_3_* is except for carbonyl oxygen.

### S5.2.2. Unsaturated bonds of carbon chain

Unsaturated bonds on backbone carbon chain are also counted as modifications. Basically, the modification counts increase as the bond order increases. Note that the unsaturated bonds are counted for each carbon on the bonds because they change the states of the carbons. Therefore, each unsaturated bond is always counted twice. **Fig.** **S5-3** shows relationships between modification counts and replacements of functional groups with a change of bond order of the backbone carbon chain from basic states.


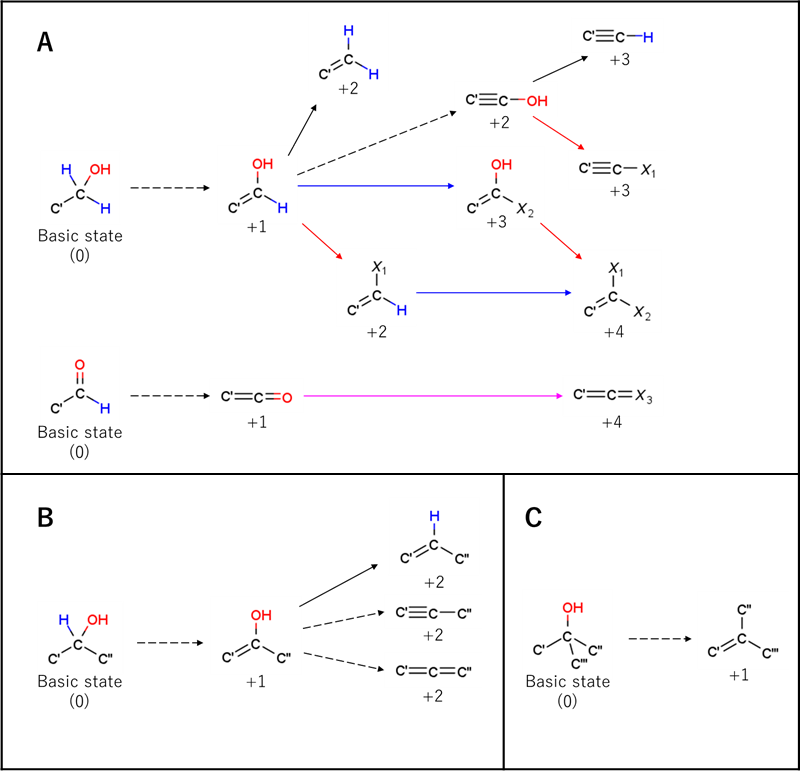


**Fig. S5-3** Relationships between modification counts and replacements of functional groups with a change of bond order of the backbone carbon chain from basic states on (**A**) terminal, (**B**) non-terminal, and (**C**) branched carbons. Each unsaturated bond on a carbon is counted as modification based on the number of the bond order (black dashed arrow, +1). The other replacements are categorized into deoxy (black arrow, +1), replacement of a hydroxyl group (red arrow, +1), replacement of a hydrogen (blue arrow, +2) and replacement of a carbonyl oxygen (pink arrow, +3). Here, *X*_1_ is a modification except for hydrogen, hydroxyl group or the derivative which hydrogen on hydroxyl group is replaced to the other modification, including *O*-glycosidic bonds. *X*_2_ is not hydrogen. *X*_3_ is modification except for carbonyl oxygen.

### S5.2.3. Rings bridging carbon on a carbon chain

Rings on a carbon chain are also counted as modification. We defined that a ring on carbon chain is a heteroatom bridging two or more carbons on a carbon chain and the modification count is the number of rings formed by the atom. For example, when one ring is formed on the carbon chain, i.e., an atom bridges two carbons, it is counted as +1. Note that the ring does not apply to cyclic hemiacetals or hemiketals because they are basic states. If an atom bridges three carbons, three rings are formed on the carbon chain and thereby it is counted as +3. Such rings can be formed when a ring is formed by an atom such as nitrogen, but the replacement is extremely rare as a modification of monosaccharide. Therefore, this counting is served as a penalty to exclude such structures far from a monosaccharide.


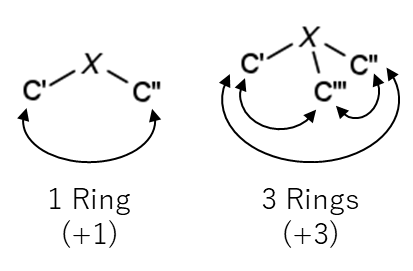


**Fig. S5-4** Relationships between modification counts and the number of rings on carbon chain. C’, C’’ and C’’’ are members of a same carbon chain.

### S5.2.4. Replacements of heteroatoms on cyclic hemiacetal/hemiketal carbon

As we mentioned above, a hemiacetal/hemiketal carbon in cyclic form is also a basic state. The hydrogen on the hemiacetal carbon cannot be replaced because it is an element of hemiacetal group, i.e., the group is not regarded as hemiacetal group when the hydrogen is replaced by the other element. On the other hand, ring oxygen and hydroxyl group (or oxygen in glycosidic bond) on the carbon can be replaced to the other elements. Thus, we also defined each oxygen replacement on the state is counted as +1 according to normal modification counting rule. There are some possible modified states for the carbon as shown in **Fig. S5-5**. Note that replacement of ring oxygen will be counted based on the state of the other carbon forming hemiacetal/hemiketal bond separately.


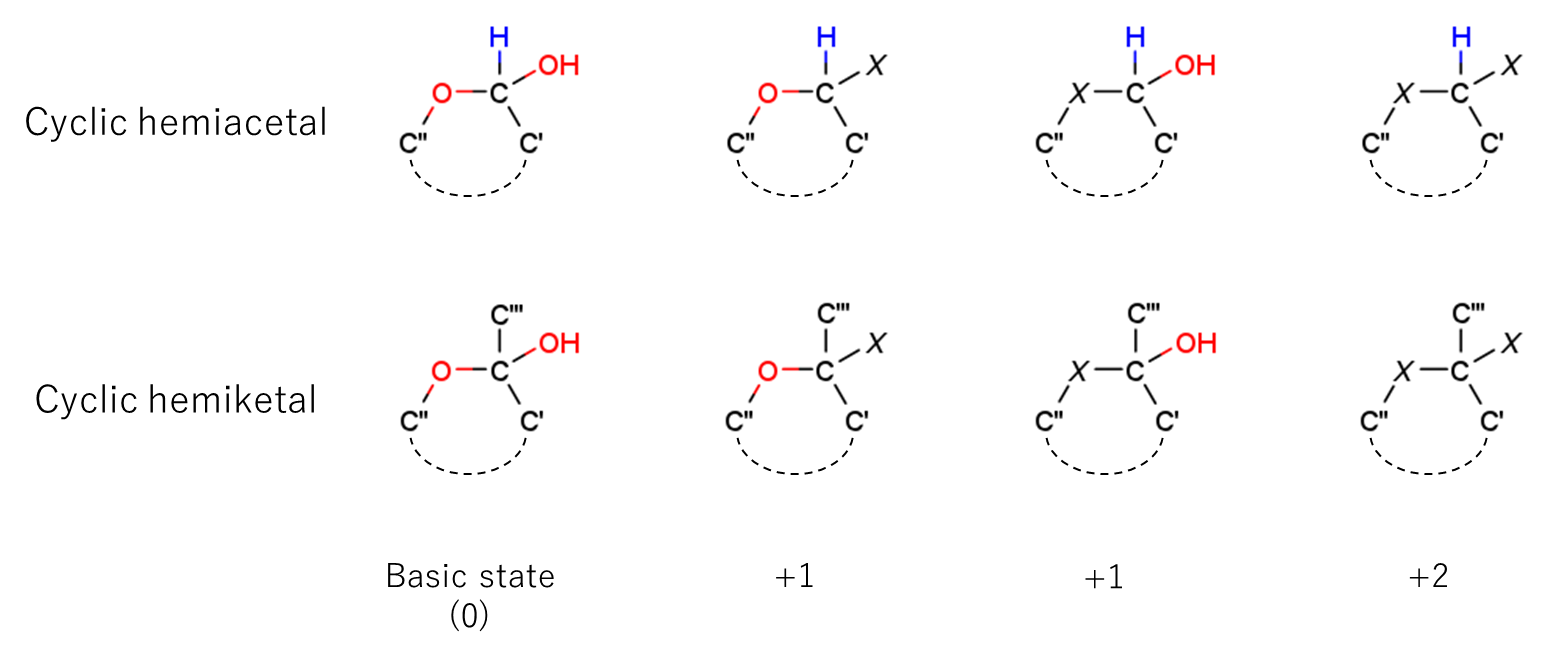


**Fig. S5-5** Relationships between modification counts and basic/modified states of cyclic hemiacetal/hemiketal carbons.

### S5.2.5. Extra (potential) carbonyl groups

The basic state of carbon chain has only one (potential) carbonyl group. Thus, we decided to count the second and latter (potential) carbonyl groups as extra modifications. Note that the term ‘potential carbonyl group’ refers to the hemiacetal or hemiketal group arising ring closure. The count is +2 because carbonyl group can be regarded as removal of a hydrogen from alcoholic carbon.

### S5.2.6. Penalty for (potential) carbonyl groups on uncommon position

Since the most of monosaccharides are aldose or 2-ketose, some kind of penalty should be imposed for any other structures. Thus, we impose +1 modification count if there is no (potential) carbonyl group at terminal (aldose) or the next carbon (2-ketose). This penalty is effective primarily against alditols.

## S5.3. Threshold value for modification counts

To exclude carbon chains far from basic state of monosaccharide using the monosaccharide count, we set a threshold value *θ*(*n*_c_) for the modification count, which is determined by the number of carbon atoms in the carbon group *n*_c_. i.e., the carbon chain is excluded when *N*_mod_ > *θ*(*n*_c_). The value for the carbon chain with typical length, 5 to 8, is *n*_c_ - 3. On the other hand, by this definition, the longer the carbon chain, the more modifications it can have. i.e., There is a risk that certain carbon chains will be considered monosaccharides simply because they are long, even though they have many modifications. Thus, we applied a fixed threshold value 5 for the carbon chains with longer length, 9 or more, as additional penalty for the longer carbon chains. These number was roughly determined to take known monosaccharides as monosaccharides.

$$\begin{aligned} \boldsymbol{\theta}\left( \boldsymbol{n}_{\text{c}} \right)\boldsymbol{=}\text{max}\left( \boldsymbol{1,}\text{min}\left( \boldsymbol{n}_{\text{c}}\boldsymbol{-3, 5} \right) \right)\boldsymbol{\#}\left( SEQ Equation \backslash* ARABIC 2 \right) \end{aligned}$$

# S6. Dataset for detection of monosaccharides with trivial names

In order to extract all of the monosaccharides with trivial names, we applied our glycan extraction algorithm to the monosaccharides listed in the Appendix of *2-Carb*. In this test, we focused on modification counting rules to confirm how the rule works to distinguish carbon chains. Here, stereoisomeric monosaccharides were treated as the same structure because our rule does not consider the stereochemistry of the structures. As a result, we assembled a dataset of 66 monosaccharides without stereochemistry. In this section, we listed structures and trivial names of the monosaccharides and the results of applications of our monosaccharide extraction rules. The monosaccharides with SNFG symbols are listed in S6.1 and the others are listed in S6.2.

## S6.1. Monosaccharides with SNFG symbols

| No. | Topology of molecular structure | Corresponding monosaccharides | Modification score and the break down |
| --- | --- | --- | --- |
| 1 | 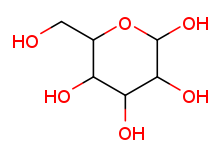 | Hex, Glc, Man, Gal, Gul, Alt, All, Tal, Ido | 0/3 |
| 2 | 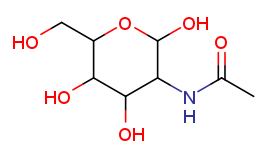 | HexNAc, GlcNAc, ManNAc, GalNAc, GulNAc, AltNAc, AllNAc, TalNAc, IdoNAc | 1/3  (N-acetyl +1) |
| 3 | 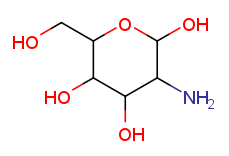 | HexN, GlcN, ManN, GalN, GulN, AltN, AllN, TalN, IdoN | 1/3  (amino +1) |
| 4 | 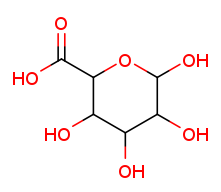 | HexA, GlcA, ManA, GalA, GulA, AltA, AllA, TalA, IdoA | 1/3  (carboxy +1) |
| 5 | 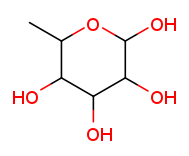 | dHex, Qui, Rha, 6dGul, 6dAlt, 6dTal, Fuc | 1/3  (6-deoxy +1) |
| 6 | 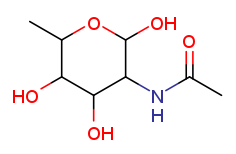 | dHexNAc, QuiNAc, RhaNAc, 6dAltNAc, 6dTalNAc, FucNAc | 2/3  (N-acetyl +1, 6-deoxy +1) |
| 7 | 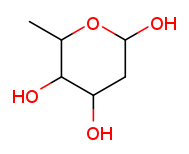 | ddHex, Oli, Dig | 2/3  (2,6-dideoxy +2) |
| 8 | 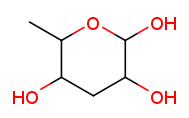 | Tyv, Abe, Par, Col | 2/3  (3,6-dideoxy +2) |
| 9 | 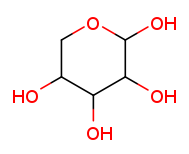 | Pen, Ara, Lyx, Xyl, Rib | 0/2 |
| 10 | 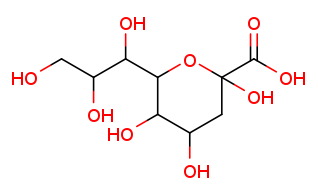 | Kdn | 2/5  (carboxy +1, 3-deoxy +1) |
| 11 | 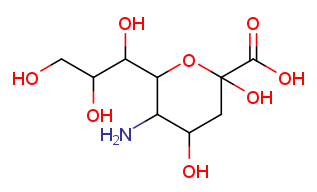 | Neu | 3/5  (carboxy +1, 3-deoxy +1, amino +1) |
| 12 | 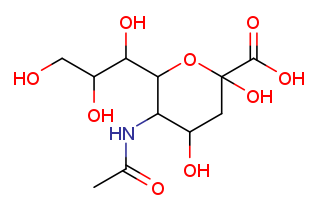 | Neu5Ac | 3/5  (carboxy +1, 3-deoxy +1, N-acetyl +1) |
| 13 | 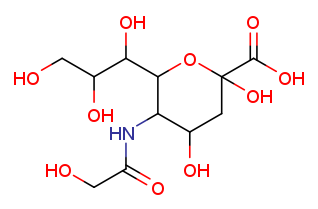 | Neu5Gc | 3/5  (carboxy +1, 3-deoxy +1, N-glycolyl +1) |
| 14 | 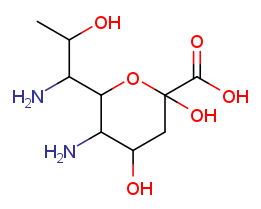 | Di-deoxynonulosonate, Pse, Leg, Aci, 4eLeg | 5/5  (carboxy +1, 3,6-dideoxy +2, 5,7-diamino +2) |
| 15 | 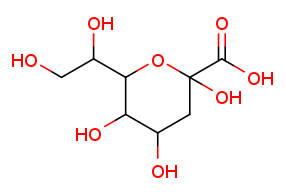 | Kdo | 2/5  (3-deoxy +1) |
| 16 | 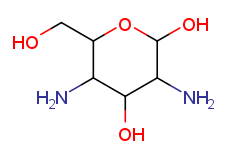 | Bac | 2/3  (2,4-diamino +2) |
| 17 | 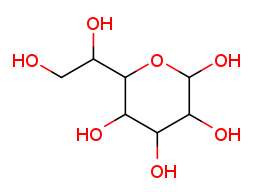 | LDmanHep, DDmanHep | 0/4 |
| 18 | 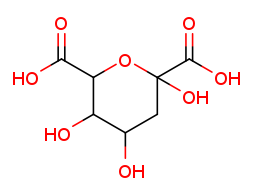 | Dha | 3/4  (dicarboxy +2, 3-deoxy +1) |
| 19 | 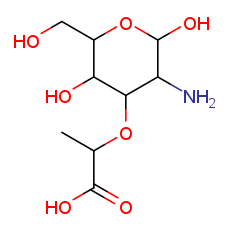 | Mur | 1/3  (amino +1) |
| 20 | 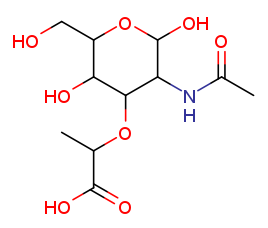 | MurNAc | 1/3  (N-acetyl +1) |
| 21 | 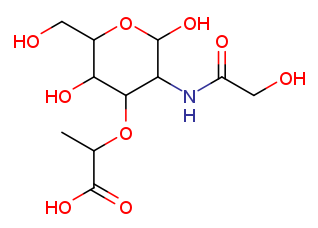 | MurNGc | 1/3  (N-glycolyl +1) |
| 22 | 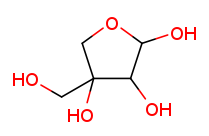 | Api | 0/1 |
| 23 | 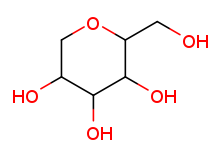 | Fru, Tag, Sor, Psi | 0/3 |

**Fig S6-1.** Structures and their modification scores of monosaccharides defined as SNFG symbols. “Sia”, “Unknown” and “Assigned” are not considered since no structures are defined.

## S6.2. Monosaccharides with trivial name (no SNFG symbol)

| No. | Topology of molecular structure | Corresponding monosaccharides | Modification score and the break down |
| --- | --- | --- | --- |
| 24 | 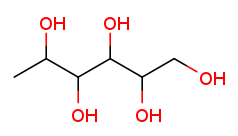  1-Deoxy-hexositol | D-Fuc-ol, L-Fuc-ol, Rha-ol | 2/3  (1-deoxy +1, No anomeric center +1) |
| 25 | 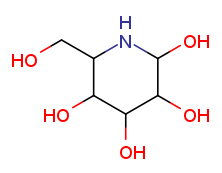  5-Deoxy-5-amino-hexose | Nojirimycin | 2/3  (5-deoxy-5-amino +1, A replacement of ring oxygen +1) |
| 26 | 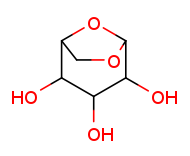  1,6-Anhydro-hexopyranose | Levoglucosan | 1/3  (1,6-anhydro +1) |
| 27 | 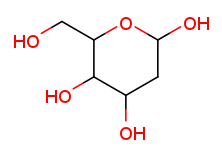  2-Deoxy-hexose | 2-Deoxyglucose(2dGlc) | 1/3  (2-deoxy +1) |
| 28 | 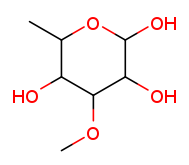  6-Deoxy-3-*O*-methyl-hexose | Digitalose | 1/3  (6-deoxy +1) |
| 29 | 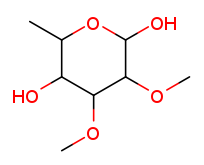  6-Deoxy-2,3-di-*O*-methyl-hexose | Mycinose | 1/3  (6-deoxy +1) |
| 30 | 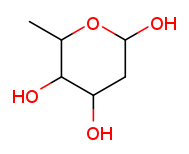  2,6-Dideoxy-hexose | Boivinose | 2/3  (2,6-dideoxy +2) |
| 31 | 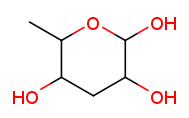  3,6-Dideoxy-hexose | Ascarylose | 2/3  (3,6-dideoxy +2) |
| 32 | 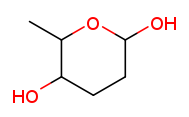  2,3,6-Trideoxy-hexose | Amicetose, Rhodinose | 3/3  (2,3,6-trideoxy +3) |
| 33 | 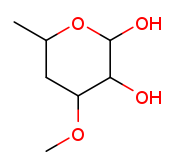  4,6-Dideoxy-3-*O*-methyl-hexose | Chalcose | 2/3  (4,6-dideoxy +2) |
| 34 | 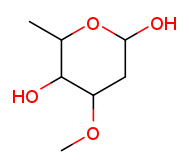  2,6-Dideoxy-3-*O*-methyl-hexose | Cymarose, Diginose, Oleandrose, Sarmentose | 2/3  (2,6-dideoxy +2) |
| 35 | 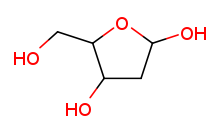  2-Deoxy-pentose | 2-Deoxyribose(dRib) | 1/2  (2-deoxy +1) |
| 36 | 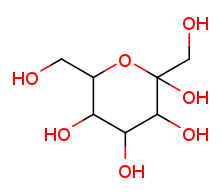  Hept-2-ulose | Sedoheptulose | 0/4 |
| 37 | 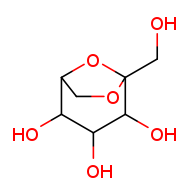  2,7-Anhydro-hept-2-ulopyranose | Sedoheptulosan | 1/4  (2,7-anhydro +1) |
| 38 | 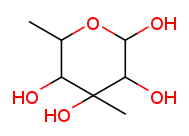  6-Deoxy-3-*C*-methyl-hexose | Evalose | 2/4  (6-deoxy +1, deoxy on 3-*C*-methyl +1) |
| 39 | 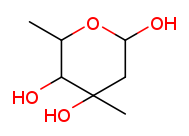  2,6-Dideoxy-3-*C*-methyl-hexose | Mycarose | 3/4  (2,6-dideoxy +2, deoxy on 3-*C*-methyl +1) |
| 40 | 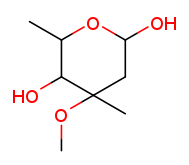  2,6-Dideoxy-3-*C*-methyl-3-*O*-methyl-hexose | Arcanose, Cladinose | 3/4  (2,6-dideoxy +2, deoxy on 3-*C*-methyl +1) |
| 41 | 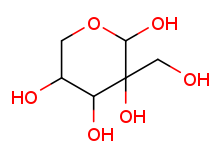  2-*C*-(Hydroxymetyl)-pentose | Hamamelose | 0/3 |
| 42 | 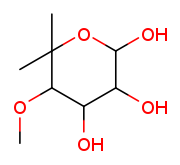  6-Deoxy-5-*C*-methyl-4-*O*-methyl-hexose | Noviose | 2/4  (6-deoxy +1, deoxy on 5-*C*-methyl +1) |
| 43 | 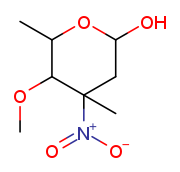  2,3,6-Trideoxy-3-*C*-methyl-4-*O*-methyl-3-nitro-hexose | Evernitrose | 4/4  (3-deoxy-3-nitro +1, 2,6-dideoxy +2, deoxy on 3-*C*-methyl +1) |
| 44 | 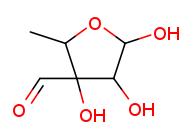  5-Deoxy-3-*C*-formyl-pentose | Streptose | 3/3  (5-deoxy +1, extra carbonyl group on 3-*C*-formyl +2) |
| 45 | 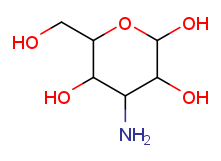  3-Amino-3-deoxy-hexose | Kanosamine | 1/3  (3-amino-3-deoxy +1) |
| 46 | 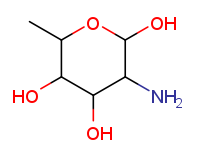  2-Amino-2,6-deoxy-hexose | Pneumosamine | 2/3  (2-amino-2-deoxy +1, 6-deoxy +1) |
| 47 | 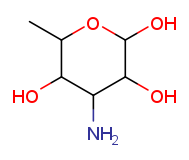  3-Amino-3,6-deoxy-hexose | Mycosamine | 2/3  (3-amino-3-deoxy +1, 6-deoxy +1) |
| 48 | 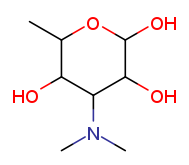  3,6-Deoxy-3-dimethylamino-hexose | Mycaminose | 2/3  (3-deoxy-3-dimetylamino +1, 6-deoxy +1) |
| 49 | 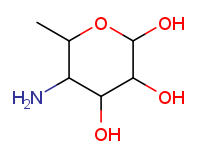  4-Amino-4,6-deoxy-hexose | Perosamine | 2/3  (4-amino-4-deoxy +1, 6-deoxy +1) |
| 50 | 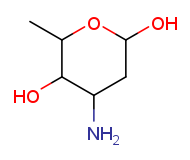  3-Amino-2,3,6-trideoxy-hexose | Acosamine, Daunosamine | 3/3  (3-amino-3-deoxy +1, 2,6-dideoxy +2) |
| 51 | 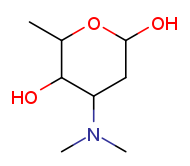  2,3,6-Trideoxy-3-dimethylamino-hexose | Rhodosamine | 3/3  (3-deoxy-3-dimetylamino +1, 2,6-dideoxy +1) |
| 52 | 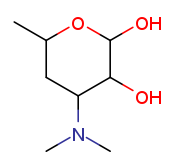  3,4,6-Trideoxy-3-dimethylamino-hexose | Desosamine | 3/3  (3-deoxy-3-dimetylamino +1, 4,6-dideoxy +1) |
| 53 | 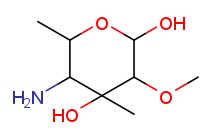  4-Amino-4,6-dideoxy-3-*C*-methyl-2-*O*-methyl-hexose | Kansosamine | 3/4  (4-amino-4-deoxy +1, 6-deoxy +1, deoxy on 3-*C*-methyl +1) |
| 54 | 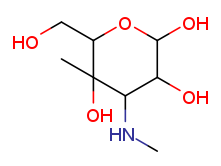  3-Deoxy-4-*C*-methyl-3-methylamino-hexose | Garosamine | 2/4  (3-methylamino-3-deoxy +1, deoxy on 4-*C*-methyl +1) |

**Fig S6-2.** Structures and their modification scores of monosaccharides with trivial name (no SNFG symbols) which have modification counts lower than the thresholds.

| No. | Structure | Trivial name | Reason |
| --- | --- | --- | --- |
| 55 | 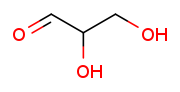  2,3-Dihydroxypropanal | D/L-glycelaldehyde | Too small |
| 56 | 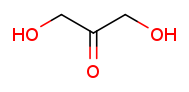  1,3-Dihydroxypropanone | Glycerone | Too small |
| 57 | 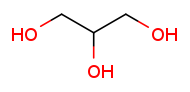  Propane-1,2,3-triol | glycerol (Gro) | Too small |
| 58 | 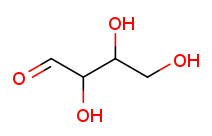  Tetrose | D/L-Erythrose, D/L-Threose | Too small |
| 59 | 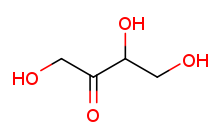  Tetrulose | D/L-Erythrulose | Too small |
| 60 | 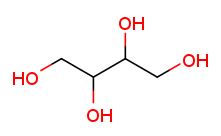  Tetritol | Erythritol, Threitol | Too small |
| 61 | 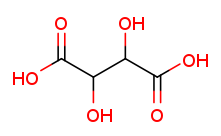  Tetraric acid | Tartaric acid | Too small |
| 62 | 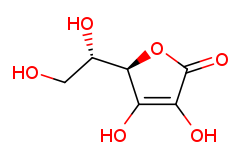  L-*threo*-Hex-2-enono-1,4-lactone | Ascorbic acid | Too many modifications  (5/3)  No anomeric center +1  1 carboxy +1  1 bridge +1  1 double bond on chain +2 |
| 63 | 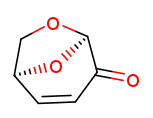  1,6-Anhydro-3,4-dideoxy-β-D-*glycero*-hex-3-enopyranos-2-ulose | Levoglucosenone | Too many modifications  (6/3)  2 deoxy  1 bridge (anhydro)  1 extra carbonyl  1 double bond on chain |
| 64 | 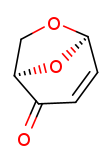  1,6-Anhydro-2,3-dideoxy-β-D-*glycero*-hex-2-enopyranos-4-ulose | Isolevoglucosenone | Too many modifications  (6/3)  2 deoxy  1 bridge (anhydro)  1 extra carbonyl  1 double bond on chain |
| 65 | 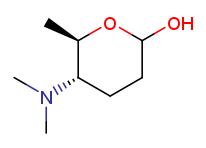  2,3,4,6-Tetradeoxy-4-dimethylamino-D-*erythro*-hexose | Forosamine | Too many modifications  (4/3)  3 deoxy  1 substitution |
| 66 | 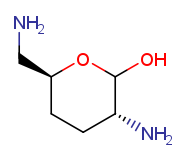  2,6-Diamino-2,3,4,6-tetradeoxy-D-*erythro*-hexose | Purpurosamine C | Too many modifications  (4/3)  2 deoxy  2 substitutions |

**Fig S6-3.** Structures of monosaccharides with trivial name (no SNFG symbols) and reason why they are not to be considered as monosaccharide.
